# Temporal network analysis in systems biology: concepts, inference, and validation

**DOI:** 10.3389/fbinf.2026.1822526

**Published:** 2026-05-12

**Authors:** Abir Khazaal, Fatemeh Vafaee

**Affiliations:** 1 School of Biotechnology and Biomedical Sciences, Faculty of Science, University of New South Wales, Sydney, NSW, Australia; 2 UNSW Biomedical AI, University of New South Wales, Sydney, NSW, Australia; 3 UNSW AI Institute, University of New South Wales, Sydney, NSW, Australia

**Keywords:** AI-predictive modelling, community detection, dynamic networks, network inference, systems biology, temporal network analysis

## Abstract

While network science provides a powerful framework for deciphering complex biological systems, static models often fail to capture the dynamic nature of cellular processes. Temporal network analysis addresses this by modelling biological relationships as time-indexed graphs, offering a more realistic representation of evolving biological interactions. But, biological data are often sparse, noisy, and heterogeneous, making temporal network reconstruction highly sensitive to modelling and preprocessing choices. This review synthesises temporal network analysis for systems biology with an emphasis on practical interpretability and trustworthy inference. We highlight how different notions of “time” (e.g., longitudinal measurements, condition/stage progression, or inferred trajectories) and different meanings of “edges” (e.g., physical interactions, statistical associations, or model-based influences) support different biological claims and therefore demand different validation strategies. Using a multi-scale perspective, we survey approaches for characterising local dynamics, tracking mesoscale reorganisation such as module and community changes, and quantifying global shifts in network topology, alongside common tasks including rewiring detection, network comparison, and community evolution. A central message is that inference is often the bottleneck, while prediction is the temptation. We therefore foreground validation and benchmarking practices needed to distinguish genuine biological dynamics from artefacts of sampling, windowing, or model class. Finally, we discuss temporal graph learning, including temporal graph neural networks. We highlight when these methods can enable meaningful forecasting in biology, and when performance is inflated by sensitivity to network construction choices or information leakage in evaluation.

## 1 Introduction

Biological systems are dynamic in ways that matter mechanistically: regulatory influences switch on and off, interaction strengths shift, and causal influence depends on when events occur. Network-based models provide a powerful abstraction for representing these systems, where nodes denote biological entities and edges encode relationships between them (Kulandaisamy et al., 2017; Nooren and Thornton, 2003; Liu et al., 2020). Yet many biological networks are still analysed as static graphs, implicitly assuming that edges are time-invariant (Bornholdt and Schuster, 2001; Newman, 2018; Newman, 2001). When time is collapsed into a single aggregate network, analyses can miss transient interactions, distort time-respecting (causally plausible) paths, and blur temporal ordering that is essential for mechanistic interpretation (Holme and Saramäki, 2012; Wu et al., 2014; Masuda et al., 2013; Li et al., 2017).

Temporal network analysis addresses this limitation by representing networks as sequences of snapshots or time-stamped interaction events, enabling the study of network evolution, temporally constrained reachability, and dynamic reconfiguration (Holme and Saramäki, 2012; Li et al., 2017; Masuda and Lambiotte, 2016; Holme, 2015). However, in biological contexts, temporal resolution is often suboptimal: sampling is sparse, destructive (particularly in single-cell assays), and heterogeneous across modalities (Greenfield et al., 2010; Palsson, 2011; Alali and Imani, 2022). This leads to a high risk of temporal aliasing, as critical short-lived events occur between sampling points. As a result, multiple incompatible temporal networks can appear plausible unless inference choices and evaluation design are treated as first-class modelling decisions (Sun and Wang, 2019; Degirmenci et al., 2020; Blonder et al., 2012; Šverko et al., 2022; Zhong et al., 2021).

This review is organised around a practical thesis: Inference is the bottleneck; prediction is the temptation. In biology, the hardest step is often not computing temporal metrics, but inferring a biologically faithful temporal network from noisy, high-dimensional, and temporally sparse measurements (Aalto et al., 2020; Pratapa et al., 2020). The field has therefore invested heavily in inference strategies that compensate for sparse sampling, including statistical enhancement (Stegle et al., 2010; Campbell and Yau, 2016; Äijö et al., 2018; Märtens et al., 2019; Newaz and Milenković, 2020; Yu et al., 2017), multi-omics integration and incorporation of prior knowledge (Subramanian et al., 2020; Sanches et al., 2024; Conard et al., 2021; Marku and Pancaldi, 2023; Liu et al., 2017; Duren et al., 2020; Stock et al., 2025; Padi and Quackenbush, 2015; Ideker et al., 2011), and model-based approaches that explicitly encode mechanistic assumptions (Mercatelli et al., 2020; Lopatkin and Collins, 2020; Ajmal and Madden, 2023; Cheng et al., 2024; Hsu and Chen, 2022; Friedman et al., 2000). However, as inference pipelines grow more complex, so does the need for validation and benchmarking: without careful checks, different window choices, priors, or model classes can produce incompatible “explanations” from the same dataset (Šverko et al., 2022; Hulovatyy et al., 2015; Caceres and Fish, 2017; Mei et al., 2025; Gauvin et al., 2022).

Recent syntheses have surveyed graph learning across single-cell omics tasks, emphasising model families and application areas, and highlighting how graph construction choices affect downstream performance (e.g., cell-cell vs. gene-gene graphs) (Li et al., 2025; Hetzel et al., 2021). In parallel, emerging work has advanced temporal inference by reconstructing trajectories under destructive sampling, for example, using optimal-transport frameworks like OTVelo, for time-stamped single-cell expression data (scRNAseq) (Zhao et al., 2025). Furthermore, new benchmark efforts such as CausalBench have made evaluation more realistic by leveraging large-scale perturbation data (Chevalley et al., 2025). What is still missing is a unified, practical framing that connects (i) what “time” and “edges” mean, (ii) how inference choices shape the network object, and (iii) how validation and leakage-safe evaluation determine which temporal stories are trustworthy. Here, we provide that bridge by treating inference and evaluation as design problems that must be settled before prediction claims can be taken seriously.

**FIGURE 1:**
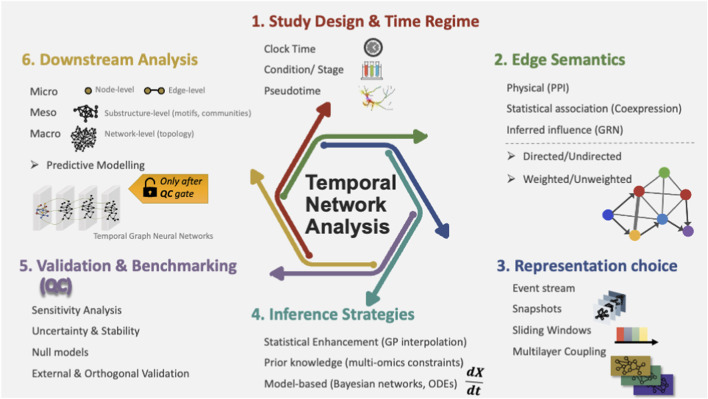
An end-to-end roadmap for temporal network analysis in systems biology. The schematic summarises the main decision points and workflow stages covered in this review. (1) Study design and time regime define what “time” represents (clock time, condition/stage, or pseudotime). (2) Edge semantics specifies what an edge means biologically (physical interactions such as protein-protein interaction networks (PPIs), statistical association such as co-expression, or inferred regulatory influence such as gene regulatory networks (GRNs), including whether edges are directed/undirected and weighted/unweighted. (3) Representation choice maps data to a temporal object (event streams, discrete snapshots, sliding windows, or multilayer coupling). (4) Inference strategies illustrate common approaches for reconstructing temporal structure from sparse/noisy measurements, including statistical enhancement (e.g., Gaussian process interpolation), incorporating prior knowledge and multi-omics constraints, and model-based formulations (e.g., Bayesian networks and ordinary differential equations (ODEs). (5) Validation and benchmarking are positioned as a quality-control (QC) gate, highlighting sensitivity analysis, uncertainty/stability quantification, temporal null models, and external/orthogonal evidence. (6) Downstream analysis spans micro-, meso-, and macro-scale characterisation (node/edge dynamics, substructures such as motifs/communities, and global topology), with predictive modelling (e.g., temporal graph neural networks) explicitly shown as appropriate only after robustness checks and leakage-safe evaluation.

To keep the review concrete and usable, we introduce two short decision Boxes early on. Box 1 distinguishes three meanings of “time” in biological network studies: clock time, condition/stage, and pseudotime; these are frequently conflated but imply different modelling assumptions and validation strategies (Ding et al., 2022; Tritschler et al., 2019; Husmeier, 2003). Box 2 defines edge semantics because the meaning of “rewiring” depends on whether edges encode physical interactions, statistical association, or inferred influence (Li et al., 2018; Zitnik et al., 2024; Oates and Mukherjee, 2012). With these foundations in place, we adopt a multi-scale perspective, examining temporal networks at the micro-scale (nodes and edges), meso-scale (substructures and communities), and macro-scale (global topology), followed by core analytical tasks and field-wide challenges. Finally, we add a dedicated validation and benchmarking section to operationalise the thesis above; inference must be made trustworthy before prediction claims can be taken seriously. Figure 1 provides an end-to-end roadmap of temporal network analysis in systems biology, from selecting a time regime and defining edge semantics to representation and inference choices. It also highlights validation and benchmarking as a quality-control gate and frames predictive modelling as appropriate only after leakage-safe evaluation and robustness checks.

Box 1 **“Time” in biological networks: three regimes with different assumptions**

A recurring source of confusion in temporal network biology is that “time” can mean at least three different things. These regimes are not interchangeable; each supports different network constructions, causal claims, and validation strategies.

1. Clock time (longitudinal time): measurements taken at known times (minutes/hours/days). This regime most directly supports temporal windows and time-respecting paths, and is essential for modelling fast signalling kinetics or time-ordered propagation. However, biological longitudinal sampling is often sparse or irregular due to experimental constraints, creating a high risk of temporal aliasing (missing transient events between samples). Window choice becomes a major modelling decision: too short yields fragmented graphs; too long averages away transient biology (Rajaguru et al., 2025; Sherwani et al., 2025; Chiappori and Cazabet, 2021; Gelardi et al., 2021).
2. Condition/stage as a proxy for time: networks indexed by disease stage, treatment phase, or developmental stage (e.g., “early vs. late”, “pre vs. post”). This is often closer to an evolving network representation: a sequence of snapshots whose spacing is not necessarily uniform. It enables useful comparative analyses (rewiring, module changes, shifting hubs) even when absolute timing is unknown, but causal interpretation must be cautious because stage labels compress heterogeneous trajectories (Upadhyaya et al., 2020; Galindez et al., 2023; Mitra et al., 2024).
3. Pseudotime (inferred latent time from snapshots): common in single-cell biology, where cells are sampled once but ordered along a trajectory. Pseudotime can create a time-like axis for network analysis, but it is model-dependent and must be treated as an inference output with uncertainty, not as ground-truth time. In practice, pseudotime is valuable for reconstructing continuous trajectories from sparse observations and can be paired with probabilistic models to propagate uncertainty into downstream analyses (Tritschler et al., 2019; Song and Li, 2021; Fang et al., 2025; Hou et al., 2023; Husmeier, 2003).

Practical implication: before building a “temporal network,” state explicitly which regime you are in. Clock time supports time-respecting paths and temporal causality; condition/stage supports snapshot comparisons and slower “evolution” narratives; pseudotime supports trajectory-aware analyses but demands uncertainty-aware validation. Throughout this review, we use “temporal network” for explicitly time-indexed interactions and “evolving network” for slower snapshot sequences, while recognising that biological studies often blend these terms.

## 2 From measurements to temporal graphs: representations and assumptions

As summarised in Figure 1, temporal conclusions often depend more on upstream design and inference decisions than on downstream metrics; we therefore make these construction choices explicit before surveying analysis methods. After defining “time” in the network, the next step is to make the network representation equally explicit, because temporal conclusions often depend more on construction choices than on the downstream metric.

### 2.1 Terminology quick map

In the literature, closely related ideas are labelled inconsistently (Holme and Saramäki, 2012; Masuda and Lambiotte, 2016; Rossi et al., 2013; Casteigts et al., 2012). We therefore use three terms with specific meanings to avoid ambiguity:

- Dynamic Network: umbrella term for any network whose topology or properties change over time.
- Temporal Network: interactions represented as discrete, time-stamped events (event sequence), enabling time-respecting paths and event-level ordering (Li et al., 2017; Holme, 2015).
- Evolving Network: slower structural change is typically analysed as a series of snapshot graphs, often widely spaced (e.g., disease stages or longitudinal sampling points) (Albert and Barabási, 2000; Yook et al., 2001; Xu and Hero, 2013).

### 2.2 From measurements to graphs

**FIGURE 2:**
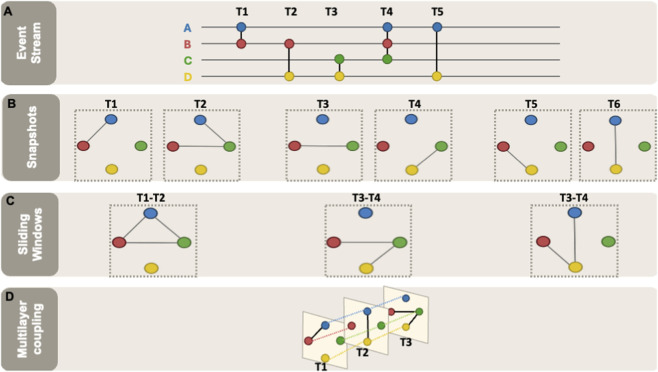
Mapping biological measurements to temporal graph objects. Four common representations are used to encode temporal information in biological networks, and each preserves different aspects of temporal structure for downstream analysis. **(A)** Event stream: interactions are represented as time-stamped events, preserving event order and enabling time-respecting paths. **(B)** Discrete snapshots: one graph per time point or condition (e.g., T1–T6), enabling comparison of topology and metrics across states. **(C)** Sliding windows: overlapping windows aggregate events/measurements within each window to produce a smoother sequence of graphs, at the cost of introducing a window-size hyperparameter. **(D)** Multilayer coupling: a stack of time-indexed layers with within-layer edges and explicit inter-layer coupling linking nodes across adjacent times, supporting temporally regularised inference and community tracking.

Given a time regime, biological data are typically mapped into one of four analysable objects (Figure 2): (i) an event stream (time-stamped interactions), (ii) a sequence of discrete snapshots (one graph per time point/condition), (iii) sliding-window snapshots (overlapping windows that smooth estimates), or (iv) an explicitly coupled multilayer model (within-layer edges plus between-layer coupling) (Li et al., 2017; Hulovatyy et al., 2015; Boccaletti et al., 2006; Moctar et al., 2019; Kivelä et al., 2014). In snapshot-based settings, the temporal window is the key knob, with overly short windows yielding sparse, fragmented graphs. In contrast, overly long windows may average away transient biology and create “rewiring” artefacts (Blonder et al., 2012; Sulo et al., 2010; Caceres et al., 2011). Because window choice is process-dependent, authors should justify it using the time scale of the phenomenon (minutes for signalling, hours/days for differentiation and months/years for progression), and, when possible, report sensitivity of conclusions to reasonable alternatives (Mittnenzweig et al., 2021; Blazek et al., 2015; Ahmad et al., 2019; Mayran et al., 2018). These choices define the temporal dynamics of the biological network and provide an explicit framework for mapping observations onto graph objects.

A second, equally consequential modelling choice is the biological definition and estimation of network edges. The significance of “rewiring” is fundamentally tied to the nature of the connection (be it a physical interaction, statistical dependency, or directed influence) (Marku and Pancaldi, 2023; Cho et al., 2004; Safari-Alighiarloo et al., 2014; Koh et al., 2012; Quach et al., 2007). We provide a taxonomy of these edge semantics in Box 2.

Box 2 **Edge semantics: functional definitions and dynamics**

A temporal network analysis is only interpretable if the edge semantics are explicit (what an edge represents biologically and how it is estimated).

- Physical interaction edges: Edges represent direct molecular interaction (e.g., protein binding in protein-protein interaction networks (PPIs). Here, an edge changing over time can plausibly indicate condition-specific complex assembly, signalling complex transitions, or context-dependent binding (Wu et al., 2024; Akbarzadeh et al., 2024; Stacey et al., 2018; Greenblatt et al., 2024). Validation can be supported by orthogonal assays or established interactome databases.
- Statistical association edges: Edges represent correlation/association (e.g., co-expression) inferred from measurements. Temporal changes then reflect shifting covariance structure, which can be biologically meaningful but is not itself causality. These edges are highly sensitive to sampling density, window choice, and noise, so stability checks and null models become essential (Van Dam et al., 2018; Lau et al., 2020; Masuda et al., 2025; Yu, 2018).
- Regulatory influence edges (model-based): Edges represent inferred influence (often directed) from model classes such as Bayesian networks, Boolean networks, ODE/state-space models, or optimisation-based inference. In this regime, an edge implies a conditional dependency or dynamical coupling under assumptions, not necessarily a directly observed interaction (Pratapa et al., 2020; Marku and Pancaldi, 2023; Bravo González-Blas et al., 2023). This is especially relevant for GRN inference from time-series transcriptomics and integrated multi-omics data (Li et al., 2024; Yuan and Duren, 2025).

Regardless of the specific edge semantics, temporal dynamics typically manifest through four primary modalities: (i) topological alterations (presence/absence), (ii) fluctuations in edge weight or strength, (iii) shifts in directionality, and (iv) changes in the effective lag or temporal order (Holme and Saramäki, 2012; Masuda and Lambiotte, 2016; Gauvin et al., 2022; Masuda et al., 2021). Consequently, the interpretation of network ‘rewiring’ is not universal; any such claim must be contextualized within the chosen edge regime (physical, statistical, or model-based) to ensure that biological conclusions and validation strategies remain consistent with the underlying data representation.

In summary, formalising the temporal regime and edge semantics provides the necessary guardrails for meaningful analysis, ensuring that observed dynamics reflect biology rather than modelling artifacts. As we have argued, solving this ‘upstream’ bottleneck of inference is a prerequisite for any trustworthy prediction. Table 1 summarises the minimum reporting items required to make temporal network results comparable and leakage-resistant across studies. Once these construction choices are stabilised and validated, the focus can shift from the mechanics of the network to its functional architecture. In the following section, we examine how these temporal characteristics manifest across biological scales.

**TABLE 1:** Minimum reporting checklist for biological temporal network studies. Items reflect common failure modes in temporal inference and evaluation (e.g., sensitivity to windowing, uncertainty in inferred time, and information leakage in prediction) and align reporting with time regime and edge semantics.

Time regime	Define whether “time” is clock time, condition/stage, or pseudotime; if pseudotime is used, describe the inference method and how ordering uncertainty is handled
Edge semantics	State what an edge represents (physical interaction, statistical association, or inferred influence); specify whether edges are directed/undirected and how weights are defined
Graph construction	Describe the mapping from measurements to graphs (event definition or windowing rule); report filtering/thresholding or regularisation; specify how missingness and batch/technical effects were handled
Inference method	Name the model class and key hyperparameters; describe priors/constraints (e.g., multi-omics or curated knowledge) and how they enter the inference
Stability/Sensitivity	Re-run inference under at least two reasonable alternatives (e.g., window sizes or smoothing strengths) and report stability summaries (edge overlap, hub rank consistency, module persistence)
Null model	Specify the temporal null model used (what temporal statistics are preserved vs. randomised) and what hypothesis it tests
External evidence	Report supporting evidence where available (enrichment against known PPIs/TF binding, agreement with orthogonal omics, or perturbation support)
Prediction evaluation	Define the prediction task; use an explicit temporal split; apply subject-/sample-level separation when relevant; document leakage checks and negative sampling choices for temporal link prediction

**FIGURE 3:**
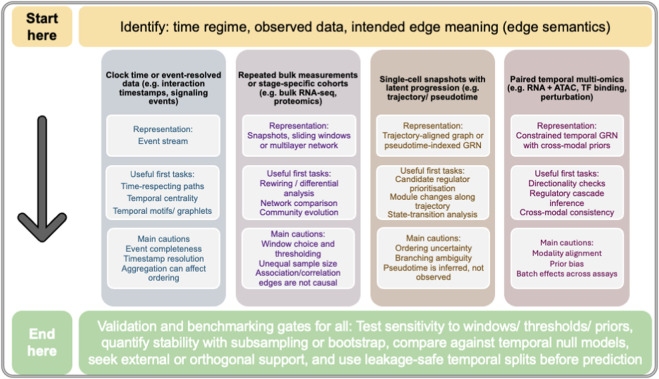
Practical decision guide for temporal network analysis in systems biology. This schematic provides a pragmatic framework for aligning common biological data settings with appropriate temporal network representations, initial analytical tasks, and key inferential cautions. The guide begins by asking the researcher to identify the time regime, the observed data type, and the intended edge meaning before selecting a downstream analytical strategy. Four common starting points are distinguished: clock-time or event-resolved data, repeated bulk measurements or stage-specific cohorts, single-cell snapshots with latent progression, and paired temporal multi-omics data. For each setting, the figure indicates a suitable representation of temporal structure, including event streams, snapshot or multilayer networks, trajectory-aligned or pseudotime-indexed gene regulatory networks, and constrained temporal regulatory networks informed by cross-modal priors. It also highlights sensible first analyses, such as time-respecting paths, temporal centrality, rewiring or differential analysis, network comparison, community evolution, candidate regulator prioritisation, state-transition analysis, directionality checks, and cross-modal consistency assessment. The lower panel emphasises that, irrespective of data type, temporal conclusions should be supported by validation and benchmarking steps, including sensitivity analyses, stability assessment by subsampling or bootstrap, comparison with temporal null models, external or orthogonal support where available, and leakage-safe temporal splits before prediction. The guide is intended to be practical rather than exhaustive and reinforces the central argument of this review: in temporal biology, method choice must remain consistent with the meaning of time, the meaning of edges, and the validation strategy used to support biological claims.

Complementing Table 1, we distil these reporting principles into a practical decision guide for common biological data settings (Figure 3), linking the type of temporal information available to the corresponding graph representation, sensible first analytical tasks, and the main inferential cautions that should be addressed before biological claims are made.

## 3 Characteristics of temporal networks across scales

Having defined the core terminology and the two decision points: what “time” represents and what an “edge” means, we now turn to analysis methods for temporal networks (TNs) in biology. In practice, many biological studies represent time as a sequence of snapshot graphs (what we call evolving networks in Section 2.1), so throughout Sections 3–5, we use “TNs” as a shorthand that includes both event-stream temporal networks and snapshot-based evolving networks unless stated otherwise. We adopt a multi-scale perspective: micro-scale measures describe node and edge dynamics, meso-scale approaches capture evolving substructures and communities, and macro-scale metrics summarise global topology and stability. We begin our exploration at the most granular level: the micro-scale.

### 3.1 Micro-scale (local patterns)

At the micro-scale, analysis focuses on the properties of individual nodes and edges, revealing the changing roles of key biological entities over time.

#### 3.1.1 Nodes

Node importance is often assessed using metrics like *degree* (number of connections) and *betweenness centrality* (influence over paths through the network). These centrality metrics can highlight influential “hubs”, whose biological significance is highlighted via the “centrality-lethality” observation in PPI networks (Jeong et al., 2001; He and Zhang, 2006). However, hub-essentiality patterns are context- and assay-dependent; in temporal settings, centrality is therefore most useful for identifying transient control points rather than universal hubs. Moreover, direct comparisons across networks are often confounded because changes in network size and density can shift centrality baselines even without biological rewiring (Zhong et al., 2022; Yu et al., 2020). In practice, robust comparison of node importance across time often benefits from normalisation or stability checks (e.g., rank-based comparisons across snapshots, and sensitivity to thresholding/resampling choices). Formal definitions of these foundational metrics are provided in Supplementary File S1, Section A.

In TNs, these measures adapt to account for time ordering. For instance, *temporal betweenness centrality* considers only time-respecting paths where interactions occur in a chronological order (Tsalouchidou et al., 2020; Zaoli et al., 2021), which is critical for modelling processes like disease transmission or signalling cascades (Machens et al., 2013; Husein et al., 2019). Such temporal metrics can reveal nodes that become transiently influential. In studies of cell differentiation, *temporal degree centrality* has been used to identify transcription factors that become *hubs* only at specific developmental stages (Xie et al., 2020). Similarly, in pancreatic ductal adenocarcinoma (PDAC), temporal centrality analysis identified distinct sets of key genes driving network stability at early (e.g., NDC80, KIF2C) versus advanced (e.g., ITGA4, ITGB4) stages of cancer progression (Pan et al., 2018); insights that would be missed in a static analysis.

#### 3.1.2 Edges

Edges in TNs represent dynamic interactions whose existence, strength (weight), and directionality can change over time. Examining these shifts can help characterise regulatory changes during biological transitions, such as immune responses or cell differentiation (Hugues et al., 2004; Sheu and Hoffmann, 2022). For instance, in GRNs, interactions emerge or vanish depending on developmental stage or environmental conditions, governing gene activation and suppression (Erwin and Davidson, 2009). Edge weights may reflect interaction intensity, like protein binding affinity or synaptic strength, that fluctuates in response to stimuli or disease progression (Lu et al., 2007). Directionality is also critical because it enables time-respecting paths, supporting models of signalling cascades or hierarchical regulation of gene expression by master regulators (Chan and Kyba, 2013; Van Der Wijst et al., 2018). Importantly, in omics-derived networks, apparent edge gain/loss is often driven by sampling, preprocessing, and inference choices; edge-level uncertainty and robustness should be assessed (and ideally reported) before interpreting rewiring as biology. A more detailed discussion of edge dynamics, including case studies on context-dependent transcription factor binding, intermittent gene co-expression, and synaptic plasticity, is provided in Supplementary File S1, Section B.

### 3.2 Meso-scale (substructures: from building blocks to functional modules)

Zooming out from individual nodes and edges, meso-scale level analysis examines subgroups of nodes whose organisation reflects intermediate-level architecture and function. Depending on the analytical questions, these substructures range from small, recurring patterns (motifs or graphlets) to maximally interconnected sets (cliques) and cohesive modules (communities). Supplementary Table S1 summarises key meso-scale substructure types, and Supplementary File S2 provides a more detailed review of formal definitions and distinctions between subgraphs, graphlets, motifs, and cliques, with a visual presentation in Supplementary Figure S2.

#### 3.2.1 Communities: bridging structure and function at a larger scale

In biological networks, rigid patterns such as motifs and cliques are often too restrictive to capture system-level organisation. Many networks instead exhibit modularity, motivating community/module discovery as a coarse-grained description of functional organisation (Arenas et al., 2008). In graph-theoretic terms, a community is not merely any subgraph, but a set of nodes whose internal connectivity is denser than expected under an appropriate null model (Supplementary Figure S3) (Radicchi et al., 2004; Porter et al., 2009; Fortunato, 2010). Numerous algorithms operationalise this idea (Coscia et al., 2011; Li et al., 2010; Ni et al., 2019), including modularity-maximising approaches that partition networks to maximise the difference between observed and expected within-community connections (Newman and Girvan, 2004; Newman, 2006; Alcalá-Corona et al., 2021). In complex disease contexts, community detection can help localise coordinated perturbations spanning multiple interacting genes or proteins, supporting mechanistic interpretation and therapeutic hypothesis generation (Sevimoglu and Arga, 2014; Valentini et al., 2014; Manipur et al., 2021).

#### 3.2.2 Meso-scale structures in TNs

The dynamic nature of real-world networks adds complexity at the mesoscale because substructures can emerge, persist or dissolve over time. In biology, temporal data are frequently analysed as snapshots (condition or timepoint-specific networks), enabling direct comparison of meso-scale structure across time (Masuda and Holme, 2019; Hosseinzadeh et al., 2022; Peixoto and Rosvall, 2017). Temporal motif analysis is most informative when interaction timing is resolved at the scale of the mechanism; in many omics-derived TNs, sparse sampling shifts emphasis toward snapshot-based module and community evolution rather than fine-grained event motifs (Kovanen et al., 2011; Liu et al., 2021; D et al., 2014; Lucas et al., 2023). This is a practical limitation rather than a dismissal, as temporal motifs can be highly informative when time-stamped interaction sequences are available, but their interpretability depends strongly on temporal resolution and event completeness.

Instead of enumerating fine-grained event motifs, many biological TN studies focus on persistence vs. transience of meso-scale structure. For example, transient clique-like assemblies can reflect time-ordered formation and disassembly of functional complexes: following stimulation, TNFR1 recruits proteins to form Complex I that drives pro-survival signalling, and subsequent disassembly enables formation of a pro-apoptotic Complex II, illustrating a temporally ordered “appearance/disappearance” of a functional substructure (Yuan and Ofengeim, 2024; Micheau and Tschopp, 2003). Tracking such patterns provides a mechanistic bridge between network dynamics and pathway logic.

**FIGURE 4:**
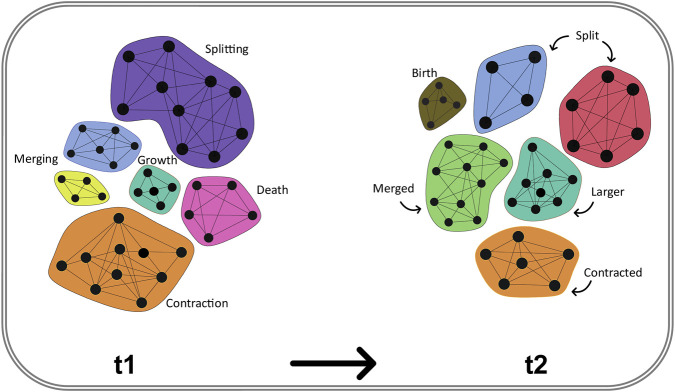
Canonical community events in temporal networks. Communities are shown at two time points (t_1_ and t_2_); colors indicate community identity across time. Examples of common temporal events include merging (two communities at t_1_ forming one at t_2_), splitting (one community at t_1_ dividing into two at t_2_), growth and contraction (node gain/loss within a community), and birth/death (appearance/disappearance of a community). These event types provide a shared vocabulary for reporting community evolution in biological temporal networks.

At a larger meso-scale, temporal community analysis focuses on events such as community growth/shrinkage and merge/split (“reorganisation”), as well as birth/death (“turnover”) (Figure 4). Complementarily, tracking intermodular vs. intramodular hubs over time can reveal whether regulation is dominated by shifting cross-module coordination or by stability within core complexes (Supplementary Figure S4) (Nooren and Thornton, 2003; Zhang, 2009; Pang et al., 2010), an axis that is often directly interpretable in disease and stress-response settings (Lin et al., 2010).

#### 3.2.3 Models for mesoscale dynamics: Markovian, higher-order, and deep learning

Capturing temporal meso-scale behaviour requires explicit modelling assumptions about how network structure evolves (Li and Maini, 2005). Markov chain models provide a simple baseline in which the state at time t depends only on recent history (first-order) or multiple prior states (higher-order), trading interpretability and statistical efficiency against the ability to represent long-range dependencies (Singer et al., 2014; Chou and D’Orsogna, 2014). However, mesoscale dynamics in biology can reflect nonlinear and delayed effects (e.g., cascades in signalling and regulation), motivating models that can learn richer temporal dependencies.

Recent deep learning approaches on temporal/dynamic graphs, such as temporal graph neural networks and temporal transformers, offer an alternative by learning time-dependent node/edge representations that can be clustered into evolving modules or used to predict future links/states (Kuhn and Oshman, 2011; Kim et al., 2018). These methods can capture complex dependencies beyond fixed-order Markov assumptions, but they introduce additional risks (overfitting, sensitivity to negative sampling/time splits, and reduced interpretability) and therefore require careful validation and reporting.

Across these approaches, comparability hinges on reporting choices that strongly shape meso-scale conclusions. We recommend reporting: (i) snapshot/window definition and effective temporal resolution; (ii) edge inference and filtering choices, including uncertainty/robustness; (iii) community/substructure method and key parameters (including temporal coupling, if used); and (iv) sensitivity of meso-scale results to reasonable alternatives (e.g., resampling, thresholds, and at least one methodologically distinct baseline).

### 3.3 Macro-scale (global properties)

While micro-scale and meso-scale analyses focus on individual components and modules, a macro-scale perspective is essential for understanding the global properties that govern the entire network’s stability, efficiency and robustness (Palazzi Nieves et al., 2019).

Classic static properties such as network density, degree distribution, clustering coefficient, modularity, and average path length provide a vocabulary for describing a network’s architecture. These measures are crucial for characterising network topology, identifying structural properties like scale-free or small-world characteristics, and quantifying functional organisation. Foundational definitions of these metrics and a visual guide are provided in Supplementary File S3 and Supplementary Figure S5.

Applying macro-scale analysis to TNs allows us to address system-level questions about how stability, resilience, and efficiency change over time. Tracking such global properties is essential for understanding large-scale biological transitions, such as developmental processes or the progression from a healthy to a disease state (Padi and Quackenbush, 2018). For example, a sharp increase in average path length (the mean shortest-path distance between reachable node pairs) over time may indicate the loss or mutation of a hub gene, disrupting network-wide structure, an effect observed in metabolic and PPI networks (Vogelstein et al., 2000; Jeong et al., 2000; Albert and Barabási, 2002). A core challenge is to adapt static metrics for dynamic topologies, and this depends directly on how time is represented (Figure 2). When interactions are available as time-stamped events (event streams), macro-scale descriptors can be defined directly in terms of time-respecting paths and temporal distances (e.g., reachability or temporal efficiency), rather than relying on a static graph that discards ordering (Masuda and Lambiotte, 2016; Holme and Saramäki, 2019).

More commonly in biological studies, time is discretised into a series of snapshots (one graph per time point/condition), and standard metrics are computed independently for each snapshot to generate trajectories that reveal global shifts over time (Holme and Saramäki, 2012; Li et al., 2017). Alternatively, a sliding window technique constructs overlapping subnetworks across successive time windows, which can yield smoother trends but introduces a window-size hyperparameter (Hulovatyy et al., 2015; Moctar et al., 2019). For sparse biological time series networks, it is therefore important to report sensitivity to conclusions to reasonable window choices (Sikdar et al., 2016; Jordan et al., 2020).

A complementary strategy is multilayer coupling, where each time point is treated as a layer, and each node is linked to its counterpart in adjacent layers. This coupling encourages continuity across time (reducing implausibly abrupt shifts caused by noise or sampling) and is widely used for temporally regularised analyses such as tracking communities or modules across layers (Kivelä et al., 2014; Mucha et al., 2010).

In all representations, metrics must be interpreted in a way that respects temporal ordering when the biological question implies ordering or propagation (e.g., signalling and regulatory cascades). However, the extent to which this is possible depends on the graph representation. When data are represented as event streams, temporal shortest paths can be defined as time-respecting paths in which each interaction occurs after the previous one, yielding chronologically plausible routes of propagation (Wu et al., 2014; George and Shekhar, 2008). Similarly, temporal clustering can be formulated in terms of causally ordered triads (triplets of nodes where interactions occur in a causally ordered sequence) or temporally valid closure patterns (Cui et al., 2013; Chen et al., 2024). By contrast, in snapshot and sliding-window representations, these quantities are usually evaluated either within each graph separately or compared across successive graphs, which captures temporal change at a coarser level but does not retain the full event ordering. In multilayer networks, related questions can be addressed by analysing paths, persistence, or community structure across coupled layers. These representation strategies are summarised in Figure 2.

An alternative modelling strategy involves TN aggregation, where a single, cumulative network, also known as a supergraph, is constructed by weighting edges based on their temporal frequency (Holme and Saramäki, 2012; Masuda and Lambiotte, 2016; Braha and Bar-Yam, 2009; Hadlak et al., 2013). While this approach summarises long-term connectivity patterns, it presents limitations (Pfitzner et al., 2013; Scholtes et al., 2016). Aggregation may obscure the timing of specific events, making it difficult to detect intermittent interactions that could be biologically or structurally significant (Cencetti et al., 2021). Furthermore, it treats all interaction frequencies equally, regardless of context (Masuda et al., 2013; Rohrschneider et al., 2010). For instance, two node pairs with identical aggregated edge weights may reflect vastly different dynamics, one arising from repeated weak interactions and the other from a few strong, temporally localised events (Blonder et al., 2012). Without careful interpretation, aggregation may overemphasise noise or underrepresent functionally significant transient events. We treat full aggregation as a baseline that discards ordering and therefore do not include it among the four time-preserving representations in Figure 2.

## 4 Analytical frontiers and core challenges in TNs

TN analysis is a broad and rapidly evolving field that is inherently complex due to the integration of time-dependent dynamics. Analytical approaches are diverse but generally aim to address a set of core objectives: tracking structural changes, quantifying similarity over time, monitoring the evolution of communities, and ultimately, building predictive models. The choice of approach typically depends on the specific analytical task and the nature of the available data.

### 4.1 Core analytical tasks

Before addressing the major challenges, it is essential to understand the primary analytical tasks that form the bedrock of TN research.

#### 4.1.1 Tracking structural changes

A core question in TN analysis is how the topology of networks evolves. Researchers investigate local and global structural changes, such as trends in network growth, fragmentation, or rewiring, which involve significant changes in node connections (Fründ, 2021; Sharma et al., 2021). In biological systems, shifts in connectivity may correspond to functional transitions, offering insights into key pathway alterations between physiological states, such as healthy vs. disease (Grimes et al., 2019; Sho and jaie, 2021). For instance, rewiring within cellular molecular networks has been implicated in driving distinct phenotypic outcomes, including embryonic development and cellular differentiation (Boland et al., 2014). To explore such dynamics, snapshot-based analysis is often used, where network metrics like degree distribution and clustering coefficient are calculated at each time point and compared across snapshots (Moctar et al., 2019; Fernex et al., 2021). Other methods include differential network analysis algorithms, which are widely regarded as effective tools for tracking topological changes and identifying recurring patterns (Zhang et al., 2022; Lichtblau et al., 2017). In parallel multilayer network models enable the study of both intra-layer edges (within a time point) and inter-layer edges (across time points) (Kivelä et al., 2014).

#### 4.1.2 Quantifying network similarity

Comparing and quantifying the similarity between networks at different time points helps assess whether a system remains stable or undergoes significant structural changes. Several methods are available for this purpose, including the commonly employed proximity statistic Jaccard Index (which measures edge overlap), graph edit distance (reflecting the minimal transformation required between two graphs), and graph kernels (functions designed to compare graph structures based on their features) (Borgwardt, 2007; Haussler, 1999). Additionally, network alignment techniques can be applied to compare networks and identify similarities. Two alignment strategies are typically used: local network alignment, which compares the building blocks of two networks, such as motifs; and global network alignment, which seeks to align entire networks by maximising structural overlap (superimposition) in a way that preserves their overall organisation (Meng et al., 2016; Milano et al., 2018). Each of these approaches constitutes a rich research domain in its own right. While a detailed discussion is beyond the scope of this review, several excellent surveys provide comprehensive overviews (Kriege et al., 2020; Guzzi and Milenković, 2018; Singh et al., 2008).

#### 4.1.3 Monitoring community evolution

This task focuses on how communities form, evolve, and dissolve within TNs. Key phenomena include community persistence, emergence, splitting, merging, and death, as outlined earlier (Figure 4). Several advanced algorithms have been adapted to capture these dynamics. For instance, the Infomap algorithm is a flow-based community detection method that tracks the evolution of communities across time (De Domenico et al., 2015). The Generalised Louvain method, on the other hand, extends modularity-based community detection by optimising both within-layer and between-layer structures (Mucha et al., 2010). Conversely, Dynamic Stochastic Block Models (DSBMs) provide a probabilistic framework to model community transitions across networks by inferring latent group structures and their evolution over time (Xu and Hero, 2013; Xu and Hero, 2014). Additional methods include identifying persistent cliques across time points (Afsarmanesh Tehrani and Magnani, 2018; Palla et al., 2007; Palla et al., 2005), detecting temporal communities via frequent pattern mining (e.g., ABACUS algorithm) (Han et al., 2007; Berlingerio et al., 2013), or modelling smooth community transitions through evolutionary clustering, which balances temporal stability with clustering quality (e.g., Facetnet framework) (L et al., 2008; Folino and Pizzuti, 2010).

### 4.2 Key challenges and future directions

While the analytical frameworks described above provide a powerful toolkit for dissecting TNs, their application to biological systems exposes a central tension: inference is the bottleneck, and prediction is the temptation. Inference asks whether we can reconstruct a temporally faithful network from sparse, noisy, and irregular measurements, whereas prediction asks whether those inferred dynamics can be used to forecast future states or responses. In practice, weak inference (e.g., unstable edges under windowing choices, uncertain pseudotime orderings, or unvalidated directionality) can propagate into confident but fragile predictive models (Alali and Imani, 2022; Blonder et al., 2012; Diaz and Stumpf, 2022). We therefore organise the remaining challenges around building defensible TN models and deploying them responsibly for prediction.

#### 4.2.1 Inference: data sparsity and temporal resolution

A primary challenge for applying TN analysis in biology stems from the nature of the data itself. Theoretical frameworks often assume high-resolution, continuous data streams, yet biological experiments are constrained by throughput, cost, and perturbation limits, resulting in coarse, irregularly sampled and incomplete datasets (Alali and Imani, 2022). This discrepancy between the ideal and the achievable can introduce significant analytical artefacts, most notably temporal aliasing.

When transient but pivotal molecular events, such as sub-second kinetics of protein-ligand binding or minutes-long phosphorylation cascades, fall between sampling points, they are missed entirely. For example, infrequent sampling of the yeast metabolic cycle can fail to capture critical oscillatory dynamics, leading to incorrect conclusions about metabolic regulation (Tu et al., 2005). Additionally, the identification of time-respecting paths is highly sensitive to missing data (Gauvin et al., 2022). In the EGF signalling pathway, for instance, sparse measurement might suggest a direct causal path (e.g., EGF → ERK), omitting the rapid intermediate events (EGF → RAS → RAF → MEK → ERK), where key proteins like MEK are active for only a few minutes (Sun and Wang, 2019; Han et al., 1993; Ambrosio et al., 1989; Marshall, 1995). Poor temporal resolution risks fundamentally misinterpreting the mechanism of signal propagation and overlooking potential drug targets (Degirmenci et al., 2020; Zhong et al., 2021). Similarly, the gradual assembly or disassembly of protein complexes, such as the exosome during cellular stress responses, can be misinterpreted as an abrupt event rather than a coordinated modular reconfiguration (Gavin et al., 2006; Gasch et al., 2000; Schlossarek et al., 2022). Mitigating these challenges represents a critical frontier, as simply increasing sampling frequency is often not practically or financially feasible.

Data-driven and statistical enhancement strategies have emerged to infer this missing information. Advanced interpolation techniques, such as Gaussian Process (GP) regression, offer a robust framework for modelling non-linear dynamics. GP regression utilises observed data points to infer intermediate states probabilistically, allowing researchers to quantify the uncertainty of these interpolations (Campbell and Yau, 2016; Märtens et al., 2019; Buettner et al., 2014). This technique has been successfully used to reconstruct gene expression trajectories in developmental biology from sparse measurements (Stegle et al., 2010; Äijö et al., 2018). Other methods, including those based on matrix factorisation or network propagation, can further enhance the interpolation by estimating the likelihood of interactions between discrete time measurements, effectively filling gaps (Newaz and Milenković, 2020; Yu et al., 2017). A distinct but equally important challenge arises with event-based data, which consists of discrete interaction timestamps rather than continuous measurements. While methods tailored for such event data are conceptually well-suited to biology due to their robustness to irregular sampling, their application has so far been limited, representing a significant area for future methodological development (Holme, 2015; Masuda and Holme, 2019; Paranjape et al., 2017).

Integrating multi-omics (e.g., transcriptomics, proteomics, epigenomics) and prior knowledge provides a complementary way to constrain network inference, reducing ambiguity (Subramanian et al., 2020; Sanches et al., 2024; Conard et al., 2021). For instance, combining chromatin accessibility data (ATAC-seq) with time-series RNA-seq can uncover regulatory cascades that are not evident from either dataset alone (Marku and Pancaldi, 2023; Liu et al., 2017; Duren et al., 2020). The accessibility data provides priors on which TFs can bind at specific times, thereby refining the interpretation of downstream transcriptional changes. Furthermore, leveraging prior biological knowledge by using known PPI networks, gene regulatory maps, or TF binding sites as structural scaffolds can guide the inference process (Stock et al., 2025; Padi and Quackenbush, 2015; Ideker et al., 2011). This approach helps to avoid biologically implausible connections that may arise from noisy time-series data, thereby yielding a more robust model of regulatory activation (Marku and Pancaldi, 2023; Chang et al., 2012).

Model-based inference represents a final, powerful paradigm that moves beyond pattern recognition toward explicit mathematical formulations of expected network dynamics, effectively encoding formal hypotheses about underlying regulatory mechanisms (Mercatelli et al., 2020; Lopatkin and Collins, 2020). Such approaches include Boolean networks, which model genes as binary switches; Bayesian networks, which use probability to identify time-consistent propagation paths; and systems of ordinary differential equations (ODEs), which describe the rates of change of molecular concentrations (Ajmal and Madden, 2023; Quach et al., 2007; S et al., 2002). Sparse experimental data are then used to calibrate these models, estimate their kinetic parameters, and select the topology that best explains the observations. Although computationally intensive, these approaches can move beyond mere correlation to a causal and predictive understanding (Cheng et al., 2024; Neapolitan, 2004). A key distinction is whether methods adopt deterministic or probabilistic frameworks to represent biology. Deterministic models (e.g., ODE systems) map the same initial condition to the same outcome, reflecting predictable dynamics (Hsu and Chen, 2022; Clermont et al., 2007). Probabilistic models (e.g., Bayesian networks) instead encode uncertainty and stochasticity, producing distributions of likely outcomes rather than single predictions (Lecca et al., 2013; Wade, 2000). In GRNs, Bayesian networks are widely used to support causal hypotheses by modelling probabilistic dependencies among genes, which is well-suited to sparse and noisy data (Friedman et al., 2000; Sachs et al., 2005). A well-parameterised mechanistic model, whether deterministic or probabilistic, can simulate the system’s response to perturbations and predict future states, fulfilling a core objective of systems biology.

#### 4.2.2 Validation and benchmarking

If inference is the bottleneck, then validation is the quality control layer that determines whether downstream temporal interpretation is biologically meaningful. Consistent with the QC “gate” in Figure 1, validation determines whether observed temporal patterns are defensible biological dynamics or artefacts of sampling, windowing, or model class. This is especially critical in temporal biology, where sparse sampling, windowing decisions, and model assumptions can produce multiple plausible, but incompatible, TNs from the same dataset. Practical validation should be matched to the time regime (Box 1) and to edge semantics (Box 2) of the TN analysis in question.

Sensitivity analysis serves as a vital first step in testing the robustness of modelling choices. Because temporal discretisation and window size can drastically reshape graph topology, hub rankings, and module structure, networks should be re-inferred under a range of window sizes or smoothing strengths (Krings et al., 2012; Bovet et al., 2022). At a minimum, researchers should report the stability of key outputs such as edge overlap and the persistence of modules (Gelardi et al., 2021; Shakil et al., 2016). For association networks, this check often distinguishes genuine rewiring from artefacts of aggregation (Cencetti et al., 2021; Rohrschneider et al., 2010).

Wherever sample size permits, uncertainty and stability should also be quantified. Techniques such as bootstrapping or subsampling can estimate confidence in inferred edges and derived features (hubs/modules), revealing whether conclusions depend on a small subset of samples (Colby et al., 2018; Langfelder et al., 2011). This is particularly relevant for pseudotime-driven analyses, where the time “axis” is itself inferred (Tritschler et al., 2019; Saelens et al., 2019). Model-based inference benefits similarly from Bayesian formulations that output distributions over parameters rather than point estimates, providing a more honest presentation of the model’s certainty (Husmeier, 2003; Golightly and Wilkinson, 2011).

Additionally, null models provide the necessary baseline for evaluating temporal claims. Unlike static null models, temporal reference models must preserve relevant structure, like event rates or inter-event times, while randomising others (Gauvin et al., 2022; Pedreschi et al., 2022; Ceria and Wang, 2023). This testing is essential for confirming whether observed dynamics, such as time-respecting paths, temporal motifs, or community evolution, exceed what would be expected from sampling noise alone. Without these constraints, missing data and discretisation are prone to creating “phantom” patterns that lack a biological basis (Smiljanić et al., 2020; Hobson et al., 2021).

Finally, external and orthogonal validation represents the highest tier of evidence, grounding inference in experimental reality (Marku and Pancaldi, 2023; Kim et al., 2023; Kamimoto et al., 2023). While partial external validation, such as enrichment of inferred edges for known PPIs or TF-binding motifs, is a common starting point for increasing interpretability, multi-omics integration provides a more rigorous filter by requiring consistency across modalities (Bravo González-Blas et al., 2023; Kernfeld et al., 2024; Wu et al., 2025; Azad et al., 2021). For example, chromatin accessibility can support or penalise TF → target edges inferred from expression (Sanches et al., 2024; Bravo González-Blas et al., 2023; Jiang et al., 2022). Moving toward causal inference, benchmarking is accelerating in settings where directionality is supported by interventions. Initiatives like CausalBench for perturbational single-cell data and Temporal Graph Benchmark 2.0 (TGB 2.0) for large-scale link prediction provide standardised metrics and reproducible pipelines for these regimes (Chevalley et al., 2023; Gastinger et al., 2024). These benchmarks allow for a clear distinction between interventional causal discovery (CausalBench) and temporal link forecasting under strict time-split evaluation (TGB 2.0). Grounding directionality in such perturbations or temporal ordering, rather than correlation alone, is essential for robust network reconstruction (Tejada-Lapuerta et al., 2025; Weinstock et al., 2024). Consequently, when translating dynamic graph learning into biology, evaluation must respect temporal ordering to avoid data leakage; at a minimum, explicit temporal splits and task definitions should be reported to ensure performance is not artificially inflated (Gastinger et al., 2024; Longa et al., 2023; Lampert et al., 2024). Such transparent protocols are particularly vital for differential network and rewiring methods, which often disagree and require comparative assessment on shared, reproducible evaluation pipelines (Galindez et al., 2023; Sharma et al., 2021). Ultimately, these validation layers convert TNs from attractive visual narratives into defensible biological models: inference becomes a claim that can be tested, rather than just a structure that can be plotted.

#### 4.2.3 Harnessing predictive power with AI

The transition from descriptive analysis and inference to predictive modelling marks the next critical frontier for the field. Predicting future network states, such as therapeutic response or disease onset, requires models capable of handling both temporal dependencies and biological complexity (Wang et al., 2018). In practice, many mature “temporal GNN” (T-GNN) deployments in biomedicine operate on patient-trajectory graphs built from electronic health records or on longitudinal brain connectivity graphs, where outcomes are directly observed (Galindez et al., 2023; Boll et al., 2024). However, when the network is inferred, predictive gains can reflect windowing/inference choices or information leakage. This leakage frequently occurs via “look-ahead bias,” where future information is inadvertently baked into the current state. For example, when a graph autoencoder uses an adjacency matrix that includes edges from the entire study duration to generate embeddings for a baseline time point (Kaufman et al., 2012). Unless evaluation explicitly separates time (using “rolling window” validation), individuals (to test inductive generalisation), and interventions, reported accuracies may be artificially inflated. Therefore, claims of forecasting must explicitly state whether the model predicts outcomes given a fixed graph, future edges/rewiring, or latent states under a mechanistic model.

T-GNNs have emerged as a particularly promising class of deep learning models for these tasks. Unlike static counterparts such as Node2vec, T-GNNs are explicitly designed to learn from evolving topologies and node features, making them well-suited for biological dynamics (Nguyen et al., 2018; Grover and Leskovec, 2016; Perozzi et al., 2014; Zhang et al., 2021). Recent applications in oncology highlight this potential: T-GNNs trained on longitudinal gene expression networks have demonstrated superior accuracy in predicting drug sensitivity compared to models relying on static snapshots (Campana et al., 2024; Nguyen et al., 2021). Such models capture the dynamic rewiring of regulatory pathways that determines a cell’s fate, offering mechanistic insights into drug response and resistance (Campana et al., 2024; Kim S. et al., 2021). Identifying disease “tipping points” represents another high-impact application of these architectures. T-GNNs can be trained on longitudinal multi-omics data to recognise the subtle network reconfigurations that serve as early-warning signals for abrupt transitions from health to disease. This approach has shown promise in the study of Alzheimer’s and other complex, progressive pathologies (Kim M. et al., 2021; Liu et al., 2019; Chen et al., 2017). However, applying these advanced AI models presents unique challenges, namely, the risk of model overfitting on small training datasets and the difficulty of capturing profound non-linearities of biological processes (Gao et al., 2023; Rajendra and Brahmajirao, 2020).

The integration of prior knowledge remains the best defence against these computational pitfalls (Kazemi et al., 2022). The most robust predictive models will therefore be those that are grounded in biological reality, using curated PPI networks or pathway databases to constrain the model’s hypothesis space (Tripathy et al., 2025; Yan et al., 2024). This not only improves predictive accuracy on limited data but also ensures the model’s outputs are mechanistically interpretable. This evolution, from descriptive metrics to robust inference and now toward AI-driven prediction, signals a paradigm shift. The convergence of time-aware network science, mechanistic modelling, and artificial intelligence promises to transform our ability to understand, predict, and ultimately control dynamic cellular systems, paving the way for precision medicine and actionable clinical insights.

### 4.3 Practical workflow guide for method selection

To consolidate the practical workflow developed across the review, Table 2 summarises representative temporal network construction, analysis, validation, and prediction families across common biological settings, together with their typical applicability, software availability, and recurrent limitations. Rather than serving as an exhaustive catalogue, the table is intended as a practical map from data characteristics to defensible methodological choices.

**TABLE 2:** Representative temporal network workflow families in systems biology**.** The table is organised into four workflow-oriented panels covering graph construction and network inference, structural comparison and mesoscale analysis, validation and benchmarking, and prediction on validated temporal graphs. It is intended as a practical, non-exhaustive guide linking common biological data settings and analytical aims to representative implementations, major strengths, and key limitations.

	Method family	Best suited to	Representative software/Implementation	Main strengths	Main limitations/Cautions
Graph construction and network inference	Event-stream temporal analysis	Directly time-stamped interactions, contact/proximity data, signaling events, or interaction logs	networkDynamic (Butts et al., 2016; Butts et al., 2014), tsna (Saqr, 2023; Saqr, 2024), ndtv (Bender-deMoll, 2024), Statnet project (Handcock et al., 2008) (R); Teneto (Thompson et al., 2017), NetworkX-Temporal (Passos et al., 2025) (Python)	Preserves event order; supports time-respecting paths, temporal centrality, and event-level motifs	Requires sufficiently resolved timestamps and reasonably complete event capture; motif results depend on temporal windowing and null choice
	Snapshot-based evolving networks	Repeated measurements at discrete time points or stage-indexed cohorts	igraph (Csardi and Nepusz, 2006) (R/Python) + custom workflow; multinet (Magnani et al., 2021) for multilayer summaries, networkDynamic (Butts et al., 2016; Butts et al., 2014), tsna (Saqr, 2023; Saqr, 2024), ndtv (Bender-deMoll, 2024), Statnet project (Handcock et al., 2008) (R)	Simple and interpretable; compatible with standard network measures and direct snapshot comparison	Sensitive to thresholding, missingness, and density differences across snapshots
	Sliding-window networks	Moderately sampled time series when smoother temporal trends are desired	NetworkX-temporal (Passos et al., 2025), Teneto (Thompson et al., 2017) (Python); custom scripts	Can reduce fragmentation and reveal gradual meso- or macro-scale change	Window size and placement can reshape topology and generate apparent rewiring artefacts
	Dynamic GRN inference with DBNs/tree ensembles/ODEs	Time-series transcriptomics, when directed regulatory influence is of interest	dynGENIE3 (Huynh-Thu and Geurts, 2018) (R/Python/MATLAB); dbnR (Quesada et al., 2025), dynUGENE (Lu and Silva, 2021) (R)	Explicit dynamical assumptions; can support directed hypotheses and perturbation-style simulation	Sample-hungry; parameter identifiability and model class strongly affect conclusions; directionality remains inferential without intervention
	Multi-omics constrained temporal GRNs	Matched or harmonised RNA + ATAC/TF-binding/perturbation data	SCENIC+ (Bravo González-Blas et al., 2023), CellOracle (Kamimoto et al., 2023) (Python); TIMEOR (Conard et al., 2021) (web/R)	Constrains biologically implausible edges and can improve mechanistic plausibility	Modality alignment, prior bias, and assay-specific noise can dominate the inferred network
	Trajectory/pseudotime-based temporal inference	Single-cell snapshots where progression is latent or only partly time-stamped	OTVelo (Zhao et al., 2025); SCENIC+ (Bravo González-Blas et al., 2023), CellOracle (Kamimoto et al., 2023) (Python); Slingshot (Street et al., 2018), Monocle 3 (Cao et al., 2019) (R)	Extracts time-like structure from snapshot data and supports candidate regulator prioritisation along trajectories	Ordering uncertainty and branch choice propagate downstream; pseudotime is not observed ground truth
Structural comparison and mesoscale analysis	Rewiring/differential network analysis	Comparisons across conditions, stages, or successive windows	DDN3 (Fu et al., 2024) (Python); DiffGraph (Gill et al., 2014), DINeR (Zhang et al., 2020) (R); custom workflows	Directly targets changing edges and condition-specific coordination	Results depend strongly on upstream network construction, and different methods may disagree
	Network similarity and alignment	Quantifying stability or change between snapshots or conditions	NetworkX (Passos et al., 2025), igraph (R/Python) (Csardi and Nepusz, 2006); GraKeL (for kernels) (Siglidis et al., 2020) (Python)	Useful for screening whether change is local or global, and for formal pairwise comparison	Different metrics emphasise different structure; edit-distance approaches can become computationally expensive
	Temporal motifs and dynamic graphlets	Event-resolved data or datasets with sufficiently fine temporal resolution	SNAP temporal motif code (Paranjape et al., 2017), dynamic graphlet research code (Hulovatyy et al., 2015); custom motif pipelines	Captures local temporal wiring patterns beyond static subgraphs	Interpretability falls when sampling is sparse; counts depend on temporal resolution and null specification
	Multilayer community detection	Layered networks where persistence and cross-layer coupling matter	multinet (Magnani et al., 2021) (R); Py3plex (Škrlj et al., 2019) (Python); GenLouvain (Jutla et al., 2011) (MATLAB)	Tracks persistent or shifting modules while borrowing strength across layers	Coupling and resolution parameters can dominate results; labels may be unstable across runs
	Dynamic stochastic block models	Networks where latent group structure and transition probabilities are of interest	dynsbm (Matias and Miele, 2017), NetMix (Olivella et al., 2022) (R)	Probabilistic treatment of community evolution with an explicit transition model	Stronger modelling assumptions and heavier computation; may underfit diffuse or non-block structure
	Frequent-pattern/clique-based community evolution	Persistence-focused analyses seeking recurring or overlapping modules	multinet (abacus, clique percolation) (Afsarmanesh Tehrani and Magnani, 2018; Berlingerio et al., 2013; Magnani et al., 2021) (R); FacetNet (L et al., 2008) implementations mostly custom/unofficial	Useful for recurrent or overlapping communities across snapshots	Can favor only highly recurring modules, miss diffuse structure, and be sensitive to support thresholds
Validation and benchmarking	Robustness, sensitivity, and uncertainty assessment	All temporal network studies, especially sparse or inference-heavy settings	Largely custom workflows; Window-size/threshold/resampling sensitivity, bootstrap or subsampling stability, uncertainty propagation for inferred time or edges CausalBench (Chevalley et al., 2023); TGB 2.0 (Gastinger et al., 2024)	Helps distinguish robust dynamics from artefacts of modelling choices, and supports leakage-safe evaluation	No single universal benchmark; validation must match time regime and edge semantics; often omitted because it is labour-intensive; does not prove correctness, but can show fragility
	Temporal null models and reference models	Testing whether temporal motifs, paths, rewiring, or community events exceed what sampling alone would produce (non-random)	Mostly custom workflows; randomised reference models preserving selected temporal constraints	Makes temporal claims falsifiable rather than purely descriptive	Choice of preserved statistics matters as much as the test itself (preserving the wrong statistics can make the test uninformative or misleading)
	Benchmark resources for inference and forecasting evaluation	Method development, comparative evaluation, and leakage-aware benchmarking	CausalBench for perturbation-based network inference benchmarking (Chevalley et al., 2023); TGB 2.0 for time-respecting future-link forecasting benchmarks (Gastinger et al., 2024)	Encourages reproducible, shared evaluation rather than one-off performance claims	Benchmark success does not automatically imply biological validity in a new dataset; benchmark and application regime must match
	External/orthogonal validation	GRN and directionality claims that need biological support beyond correlation	Modality-specific support (perturbation evidence, matched chromatin accessibility, TF-binding or prior biological evidence); generally custom integration workflows	Provides the strongest support for biological plausibility	Evidence is often partial and context-specific; lack of support is not always direct falsification
Prediction on validated temporal graphs	Temporal graph learning/temporal graph neural networks T-GNNs	Forecasting future links, node states, or graph-level outcomes from a validated temporal graph object	Temporal Graph Networks, temporal graph embedding, temporal GNN libraries such as PyTorch Geometric Temporal (Rozemberczki et al., 2021) (Python)	Can capture complex nonlinear temporal dependencies and interactions that are difficult to specify manually	Performance can be inflated by risk of data leakage, overfitting, and sensitivity to graph construction choices; often less interpretable than mechanistic models
	Mechanistic or perturbation-oriented forecasting on inferred regulatory graphs	Questions about regulatory response, intervention effects, or likely consequences of perturbing nodes in a validated GRN	Dynamic Bayesian/ODE (Quach et al., 2007)/state-space simulation, *in silico* perturbation frameworks such as CellOracle (Kamimoto et al., 2023)	Often biologically interpretable and hypothesis-generating; closer to intervention logic than black-box forecasting	Forecast quality is bounded by the validity of the inferred network and the mechanistic assumptions built into the model

Software examples are representative rather than exhaustive. Where no dominant package exists, the table states this explicitly to avoid overstating standardisation in the field.

## 5 Conclusion

Static network analysis has provided foundational insights into biological organisation, but many core biological phenomena are better understood as processes unfolding on networks rather than properties of a single fixed graph. Incorporating time reveals that biological function is encoded not only in connectivity, but in how connectivity is reconfigured: edges emerge and disappear, modules reorganise, and causal influence depends on temporal order. A multi-scale temporal perspective, from nodes and edges (micro), to motifs and communities (meso), to global topology (macro), offers a more faithful lens on mechanisms such as signalling, differentiation, and disease progression.

A unifying message of this review is captured by the thesis: inference is the bottleneck; prediction is the temptation. The central constraint in biological TNs is not the lack of analytical tools, but the mismatch between idealised high-resolution temporal models and the sparse, noisy, and irregularly sampled data available in practice. This mismatch can yield temporal aliasing, incomplete time-respecting paths, and ambiguous rewiring narratives if inference assumptions are not made explicit. The most robust path forward integrates complementary strategies: statistical enhancement for sparse sampling, biologically constrained inference using multi-omics and prior knowledge, and mechanistic model-based approaches that elevate TNs from correlation structures toward causal hypotheses.

At the same time, the field is being pulled toward prediction. Temporal graph learning and T-GNNs offer powerful frameworks for forecasting outcomes such as drug response trajectories or impending disease transitions, and early applications demonstrate clear promise (Kazemi et al., 2022; Besharatifard and Vafaee, 2024). However, predictive modelling in biology is especially vulnerable to overfitting, dataset bias, and evaluation artefacts, making validation and benchmarking essential rather than optional. Trustworthy temporal biology will increasingly depend on explicit edge semantics, explicit time regimes, and transparent evaluation protocols that respect temporal ordering and biological plausibility.

Looking ahead, the most impactful advances will likely come from tighter coupling between inference, validation, and prediction: models that integrate priors and multi-omics constraints to remain interpretable, quantify uncertainty, and generalise across cohorts and contexts. Achieving this will require sustained collaboration between experimentalists and computational scientists, and a continued shift from “networks that look dynamic” to dynamic network models that withstand falsification and support intervention.
