# Supplementary material for: Temporal network analysis in systems biology: concepts, inference, and validation
Source: Front Bioinform. 2026 May 12;6:1822526. doi: 10.3389/fbinf.2026.1822526 (PMC13200828; doi:10.3389/fbinf.2026.1822526)
Supplement: Supplementary file 1 [file DataSheet1.pdf]

Supplementary Information | Temporal Network Analysis in Systems Biology: Concepts, Inference, and Validation

Abir Khazaal[1,2,3], Fatemeh Vafaee[1,2,3]

[1] School of Biotechnology and Biomedical Sciences, Faculty of Science, University of New South Wales, Sydney, NSW 2052, Australia

[2] UNSW Biomedical AI, University of New South Wales, Sydney NSW 2052, Australia

[3] UNSW AI Institute, University of New South Wales, Sydney NSW 2052, Australia

Corresponding Author: Prof. Fatemeh Vafaee

T: +61 (2) 9065 2699 | **E**: f.vafaee@unsw.edu.au

Keywords: Temporal Network Analysis, Dynamic Networks, Systems Biology, Community Detection, Network Inference, AI-Predictive Modelling

Supplementary File 1: Foundational Micro-scale Network Metrics

Section A: Nodes

Degree

A node’s *degree* refers to the number of edges connected to it. In Gene Regulatory Networks, this represents the number of genes interacting with a given gene (node). Generally, a higher *degree* indicates greater node importance [1]. In TNs, a node’s *degree* can vary, allowing researchers to track changes in node connectivity over time and observe whether a node is gaining or losing influence. Two categories of “influential” nodes can be distinguished: those essential within a specific network at a given time point and those that remain indispensable across multiple networks over different time points [2].

Centrality

Another important class of metrics is *centrality*, which assesses a node’s importance within the network. Several types of centralities can be measured. *Degree centrality*, closely related to the concept of *degree*, is calculated by dividing the total number of edges connected to a node by the maximum possible number of connections, normalising the value to allow for meaningful comparisons across networks [3]. This measure identifies *hubs* - nodes that are critical for maintaining network stability. *Degree centrality* is particularly important because it reflects how robust a network is through the presence of these *hubs* [4]. *Betweenness centrality* is another well-studied measure that defines a node’s importance by evaluating how often it lies on the shortest paths between other nodes [5,6]. The shortest path (also known as geodesic distance) is the path with the fewest edges between two nodes [7–9].

Other approaches

Beyond traditional centrality measures, additional network metrics have been introduced to identify a minimal set of key nodes that exert full control over the network, offering significant potential in identifying disease-associated genes and drug targets [10,11]. Unlike centrality-based approaches that assign importance based on connectivity, these methods focus on structural controllability by determining the smallest subset of nodes (minimum dominating set) required to influence the entire network [12]. In breast cancer, drug target genes have been identified using an optimisation framework designed to capture dominant key regulatory nodes, demonstrating the effectiveness of such approaches in uncovering critical therapeutic targets [13,14].

Section B: Edges

Context-Dependent Interactions: The PU.1 Transcription Factor

A notable example of context-dependent edge dynamics is the PU.1 gene, a lineage-determining transcription factor (TF) critical for macrophage and B cell development[15]. PU.1 regulates gene expression by binding to distinct enhancer elements in a cell-specific manner. In macrophages, it interacts with C/EBPs, AP1 and IRF8, whereas in B cells, it associates with TFs EBF1, E2A, and IRF4. These dynamic interactions illustrate how TF networks are highly context-dependent, with PU.1's regulatory edges shifting based on the cellular environment (Fig S1) [16,17]. This highlights the limitations of static networks, which fail to capture such context-driven changes. Similarly, in PPI networks, the interaction between proteins fluctuates in response to environmental stimuli or cellular states [18]. Analysing these time-dependent changes enables researchers to identify critical periods of interaction, contributing to system-level processes such as cell differentiation, immune response, or disease progression [19].

Dynamic Edge Weight: From Protein Affinity to Synaptic Plasticity

In Gene Regulatory Networks, edge weights may indicate the strength of regulatory influence, while in PPI networks, they often correspond to binding affinity. High-affinity interactions are typically crucial for stable complexes, such as enzyme-substrate binding or receptor-ligand interactions [20]. In contrast, low-affinity interactions often play roles in transient or reversible processes, including signalling pathways or the dynamic assembly of protein complexes [21]. Notably, affinity may increase during stress responses, such as heat shock or infection, and decrease under normal conditions [22,23]. In neurobiological networks, synaptic connections between neurons exhibit changes in synaptic strength (edge weight) due to synaptic plasticity during the learning process [24,25]. Conversely, in neurodegenerative diseases like Alzheimer's, edge weights gradually weaken as synapses are lost [26]. These dynamic shifts in synaptic strength emphasise the role of TN analysis in understanding learning, memory, and neuronal transmission efficiency, particularly as they change with age.

Temporal Patterns of Edge Activity

Edges in TNs can display continuous activity, intermittent periods of inactivity, or periodic alternations between activity and inactivity at specific time points or intervals [27,28]. In a temporal gene co-expression network, for example, genes can exhibit similar expression patterns only under specific conditions, such as in response to external stimuli. Such mechanisms have been observed in yeast studies [29]. A related example can be found in immune response networks, where interactions between immune cells, such as T cells and B cells, are only activated during specific phases of infection or inflammation, reflecting the episodic nature of immune responses [30]. Understanding the temporal dynamics of these interactions, when and why they become active or inactive, offers deeper insights into immune signal transduction and gene expression regulation.

Importance of Directionality and Time-Respecting Paths

Directed edges often play a more crucial role in TNs than in static networks. When analysing connectivity and reachability in directed TNs, sometimes referred to as scheduled networks, the concept of a time-respecting directed path becomes essential [31]. This path consists of

edges with time labels that either remain constant or increase, ensuring connections progress in a biologically meaningful temporal sequence [32]. This concept is particularly significant in biological interactions, such as in GRNs, which are inherently directional [33]. Although all cells share the same genetic information, their structural characteristics and function are determined by the specific set of genes activated by other genes, known as regulators, often TFs. This directional and sequential activation enables us to differentiate master regulators, key genes at the top of the hierarchy, that initiate the cascade of gene interactions essential for cell differentiation and developmental transitions.

Supplementary File S2: A Glossary of Meso-scale Substructures

Subgraphs: The Foundational Concept

A subgraph is the most general term for a subset of a larger network, consisting of selected nodes and the edges between them. In network science, these are often called subnetworks and can range from a single node to the entire graph [35,36]. Subgraphs can be categorised as *induced*, meaning they include all edges present in the original graph between the selected nodes, or *non-induced* (partial), which include only a subset of those edges [37]. They are considered a foundational concept, with some researchers proposing *subgraph centrality* as a measurement to characterise and rank nodes. Notably, *subgraph centrality* has been observed to follow a power-law distribution, even in cases where the degree distribution of nodes does not [38,39]. This foundational concept of the subgraph allows for the definition of more specialised structures (Fig S2). One of the first refinements is to move from arbitrary subsets of a network to analysing the complete set of all small, induced subgraphs, which are known as graphlets.

Graphlets: Characterising Local Topology

Moving from the general to the specific, graphlets are small, connected, induced subgraphs, typically consisting of 2 to 5 nodes, that are used to uncover structural features of a network [40]. Unlike simply identifying the presence of certain subgraphs, graphlet analysis is often used to create a structural "fingerprint" of a network by counting the frequency of these substructures [41]. This method allows for a more detailed characterisation of a node's neighbourhood than what is possible with simpler metrics. For instance, in early analyses of PPI networks, researchers utilised a node’s *graphlet degree*, a vector counting the types of graphlets a node is part of, instead of relying solely on its *degree*. This approach enabled deeper insights into predicting protein function based on the structural context within the network [42]. Demonstrating the broader applicability of this technique, graphlet analysis has also been applied to effective brain networks, revealing differences in local structures between excitatory and inhibitory circuits and offering valuable insights into neural connectivity patterns [43].

Network motifs: Statistically Significant Patterns

While graphlets provide a complete inventory of all small local structures, network motifs are a special class of graphlets that are over-represented in a network compared to what would be expected in a random network [44,45]. This statistical significance is key; their frequent appearance suggests they have been conserved through evolution to perform specific functional roles, making them the fundamental building blocks of many biological systems [46,47]. Transcription networks, which consist of interactions between TFs and their target genes, provide a prime example of this principle [48]. Motifs initially identified in the transcription network of *Escherichia coli* have been found to share structural and functional similarities with those in diverse species, including yeast [49], plants [50], and humans [51], underscoring their fundamental role in gene regulation [52]. In directed networks, different types of motifs can be distinguished by their configuration, such as the feed-forward loop (FFL) [53] and feed-back loop [54] for three-node motifs, as well as bi-fans [55], bi-parallels [56], and other configurations for motifs involving four or more nodes [57–59]. Numerous methods and algorithms have been developed to detect, enumerate, and cluster motifs into

higher-order structures, facilitating the efficient analysis of large and complex networks [60]. While the significance of motifs is rooted in their statistical over-representation, other topological substructures, such as cliques, offer complementary insights based on deterministic connectivity patterns.

Cliques: Substructures of Maximal Connectivity

Embodying this principle of deterministic connectivity, a clique is a special type of subgraph in which every node is connected to all other nodes [61]. Defined as maximally connected subgraphs, a clique of n nodes contains $n(n-1)/2$ edges, representing maximal connectivity [62]. Originally prominent in social sciences for studying tightly linked groups (e.g., friendships), cliques have since been adopted across many fields [63,64]. In large networks, the number of cliques grows exponentially, making their identification and enumeration computationally challenging. This has led to the development of Maximal Clique Enumeration (MCE), which focuses on identifying all maximal cliques in a graph [65]. A maximal clique is a complete clique that cannot be extended, in other words, it is not a part of a larger clique [66,67]. MCE has become essential in studying complex networks, with many algorithms implemented to address it [68,69]. In biology, maximal cliques can represent sets of proteins physically or functionally connected in PPI networks or pinpoint groups of genetic loci co-regulating a particular trait or disease phenotype in quantitative trait loci (QTL) mapping [70,71]. Collectively, graphlets, motifs, and cliques represent fundamental network substructures that help reveal connectivity patterns at different scales of organisation (Fig S2). However, the strict requirement of maximal connectivity in cliques, or the small, prescribed nature of motifs and graphlets, limits their utility for describing the broader modular architecture of biological networks. This limitation necessitates methods that can identify larger, more loosely connected functional units.

Supplementary File S3: Macro-scale Metrics

In this system-wide view, we shift the focus from local patterns to holistic metrics that characterise the entire network's topology and architecture. These metrics provide critical insights into structural integrity, the potential for information flow, and system-wide resilience to perturbations [72,73].

Network Density

One of the most fundamental of these global measures is Network Density. Formally, density is the ratio of the number of existing edges (E) to the maximum possible number of edges in a network with N nodes, thereby quantifying its overall connectivity [74]. The calculation differs slightly for undirected and directed graphs. For an undirected graph, the formula is D = 2E / [N (N-1)], whereas for a directed graph it is D = E / [N(N-1)] [75]. Higher density indicates a tightly interconnected, cohesive network where information can propagate easily. Conversely, a low density suggests a sparse network, which may be more fragmented or modular (Fig S5-A) [76,77]. For example, many PPI network clustering algorithms leverage density as a guiding principle to identify functional modules. They often initiate with 'seed' proteins and expand clusters by iteratively adding neighbours, but only if the resulting subgraph maintains a predefined density threshold, thus ensuring the identified module is a tightly-knit community [78,79].

Degree Distribution

The degree distribution describes the statistical distribution of node degrees (p3), the number of connections each node has [80]. Understanding degree distribution helps determine whether a network exhibits scale-free properties, a defining characteristic of many real-world systems [81,82]. In scale-free networks, the degree distribution follows a power law, where a few highly connected nodes (hubs) coexist with many sparsely connected nodes (Fig S5-B) [83,84]. This structure has profound implications: scale-free networks are highly resilient to random failures, as removing many randomly selected nodes often leaves the overall network integrity largely intact [85,86]. However, they are highly susceptible to targeted disruptions; removal of key hub nodes can cause the entire network to collapse, a phenomenon known as attack vulnerability [87,88]. For example, in genetic networks, this structure suggests that certain key genes play central roles in cellular function. While random gene mutations may have minimal effects, losing a critical hub gene could trigger system-wide failures, leading to severe phenotypic consequences [89].

Clustering Coefficient

The Clustering Coefficient measures the tendency of nodes to form tightly knit groups or interconnected triads [90]. This metric exists at both local and global levels. The local clustering coefficient evaluates how interconnected a specific node's neighbours are, while the global clustering coefficient, more relevant at the macroscale level, captures the overall prevalence of triangular patterns, reflecting network cohesiveness [91,92]. High global clustering often signifies modular or community-based organisation within a network, whereas low clustering suggests random-like connectivity patterns [77].

Modularity

Modularity(Q) quantifies how well a network can be divided into distinct communities or modules [93]. It is a scalar value between -1 and 1 that measures the density of links inside

communities as compared to links between communities in a random network with the same degree distribution [94,95]. A positive Q indicates the presence of community structure. Networks with high modularity exhibit well-defined clusters with dense intra-community connections and sparse inter-community links. Conversely, networks with low modularity behave more like random graphs, often exhibiting degree distributions that approximate a Poisson distribution (Fig S5-C) [96,97].

Average Path Length

Average Path Length (APL) is the mean of the shortest paths between all node pairs within a network [98]. Widely recognised and frequently cited in network analysis, this metric is often termed Characteristic Path Length in social network research [99]. APL offers valuable insights into the efficiency of information, signal, or influence propagation throughout a network. For instance, in studies of neuropsychiatric disorders like schizophrenia, genes identified via genome-wide association studies (GWAS) can be mapped onto a human PPI network [100,101]. It has been observed that sets of disease-associated genes frequently form modules with a significantly shorter APL compared to randomly selected gene sets, suggesting they are functionally related and involved in common pathways [102,103]. Therefore, analysing APL can help in prioritising candidate genes from large GWAS datasets for subsequent experimental validation, ultimately deepening our understanding of disease mechanisms [104].

All these metrics provide valuable insights into the structural and functional organisation of networks. As an example, neuronal networks in the brain have been shown to exhibit small-world property, characterised by a high clustering coefficient (local connectivity within brain regions), short average path length (efficient global communication), and modularity (specialised functional regions with selective long-range connections) [105,106]. These properties collectively ensure computational efficiency and adaptability. Additional macroscale metrics, such as Assortativity (degree correlation), Betweenness Centrality distribution, and Network Diameter, as well as properties like resilience, further contribute to characterising network structure; however, they fall beyond the scope of this review.

Supplementary Figures

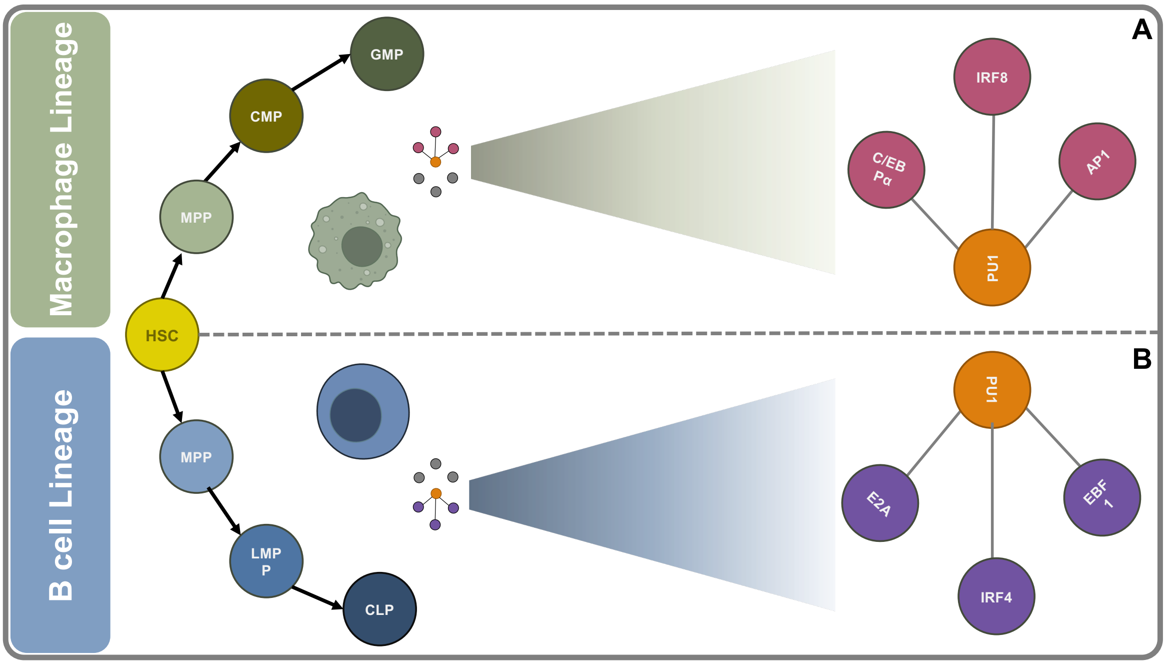

Fig S1. Context-Dependent Rewiring of the PU.1 TF Network in Macrophage and B-Cell Lineages. The figure illustrates the dynamic shift of PU.1 interactions (edges) in distinct hematopoietic progenitor cells. **A** In the macrophage lineage, within a Granulocyte Monocyte Progenitor (GMP), TF PU.1 actively interacts with C/EBPα, AP1, and IRF8 to drive the myeloid fate. Key B-cell TFs (EBF1, E2A, IRF4) are depicted as inactive (greyed out), signifying their non-functional role in this context. **B** Conversely, in the B-cell lineage, within a Common Lymphoid Progenitor (CLP), PU.1's regulatory network is rewired. It disconnects from the macrophage-specific TFs and associates with EBF1, E2A, and IRF4. TFs (C/EBPα, AP1, IRF8) are shown as inactive (greyed out). This context-driven shift in interactions highlights the dynamic nature of TF networks in determining cell fate.

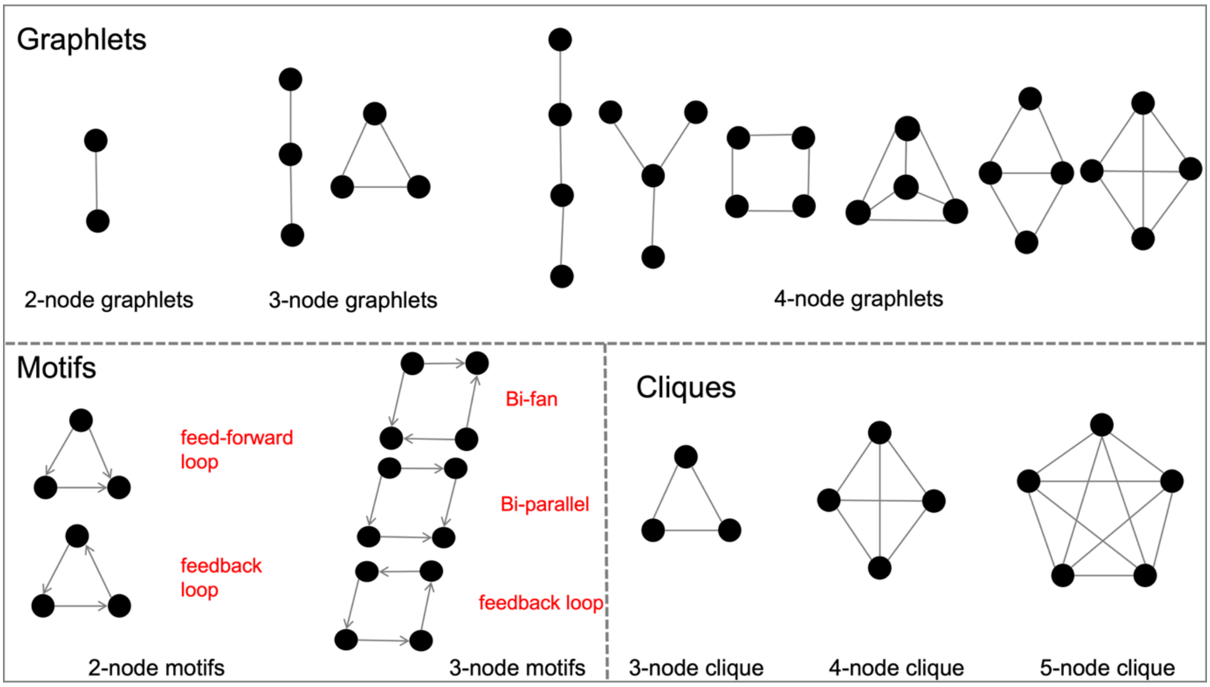

Fig S2. Graphlets, Motifs, and Cliques: Fundamental Building Blocks of Network Topology. Top: Graphlets are small, connected, and non-isomorphic subgraphs that capture local connectivity patterns. Examples of 2-node, 3-node, and various 4-node graphlets are shown. **Bottom left**: Motifs are recurrent, statistically significant subgraph patterns that often have functional implications in biological and technological networks. Common motifs such as feed-forward loops, feedback loops, bi-fan, and bi-parallel structures are depicted. **Bottom right:** Cliques are fully connected subgraphs where each node is directly linked to all others. Examples of 3-node, 4-node, and 5-node cliques illustrate increasing structural complexity.

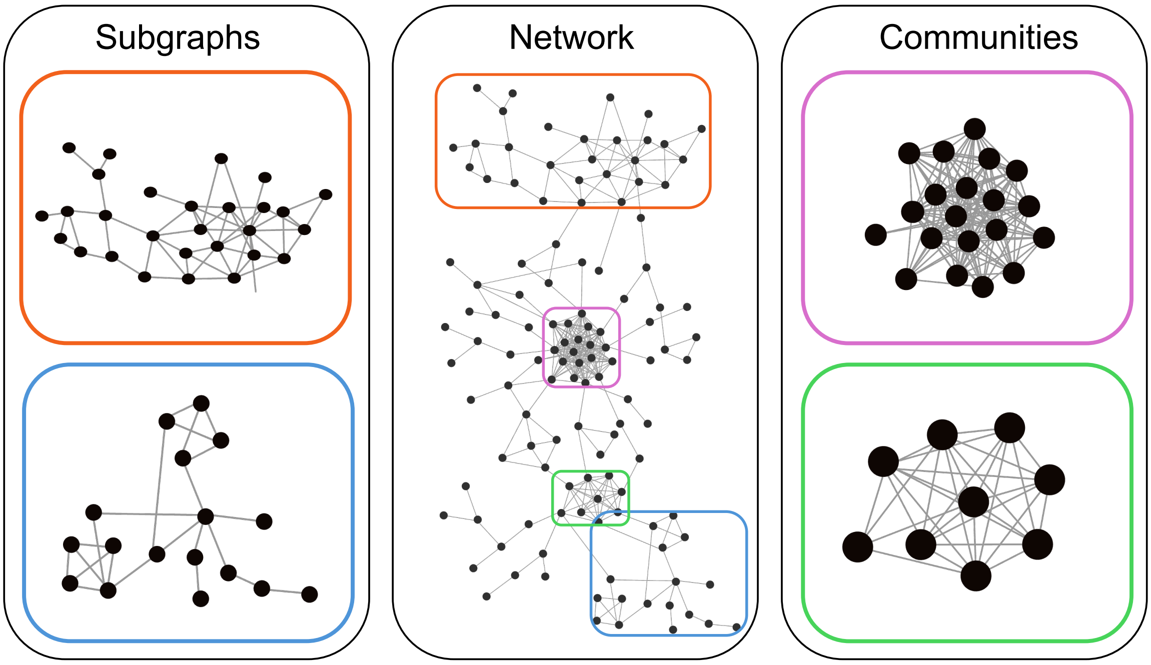

Fig S3. Network substructures, distinguishing between subgraphs and communities. A network can be analysed at different scales, revealing meaningful substructures. **Middle-Panel**: Network representation shows the overall connectivity of nodes and edges. **Left-Panel**: Subgraphs are smaller, connected subsets of the network, capturing localised interactions or functional modules. Two examples of subgraphs are highlighted (orange and blue). **Right-Panel:** Communities are densely connected groups of nodes within the network, often corresponding to functional or organisational units. Two communities with varying densities are depicted (purple and green).

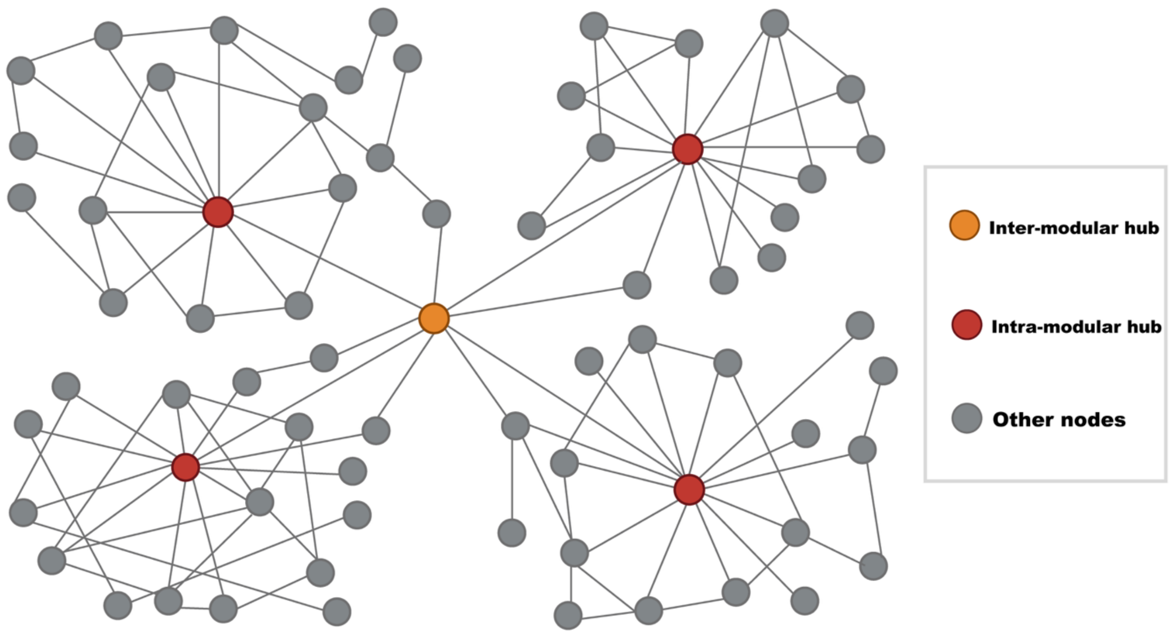

Fig S4. Intermodular vs. Intramodular Hubs. A network composed of four scale-free modules. Red nodes represent intra-modular hubs, defined as the highest-degree nodes within each module, responsible for maintaining local cohesion and function. The orange node indicates an inter-modular hub connecting to all modules' hubs and other nodes, serving as a bridge between otherwise distinct communities. Such intermodular hubs are important in TNs, where their appearance or disappearance across time points can signal critical changes in network-level communication, regulatory coordination, or system-wide transitions. All other nodes are shown in grey.

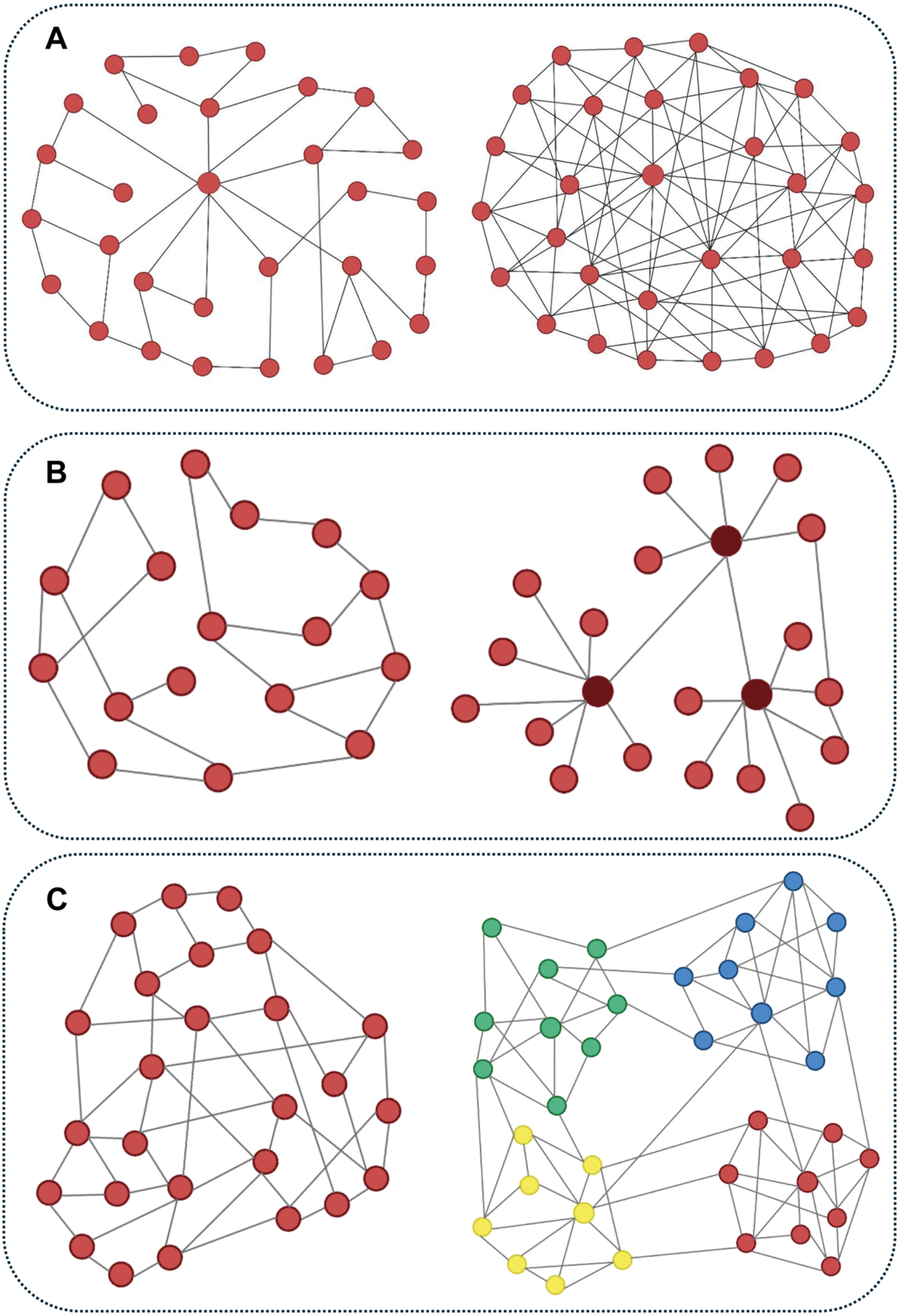
A
B
C

Fig S5. Structural properties of networks: density, topology, and modularity. **A**: Network density comparison: The left panel depicts a low-density network, while the right panel illustrates a high-density network. **B**: Network topology: The left panel shows a random network, whereas the right panel presents a scale-free network, with darker nodes indicating high-degree hubs. **C**: Modularity differences: The left panel represents a low-modularity network, while the right panel displays a high-modularity network. Node colors correspond to distinct communities, highlighting modular structure.

Supplementary Tables

Table S1 A comparative overview of distinct meso-scale network substructures, highlighting their definitions, analytical purposes, and common applications.

Substructure	***Subgraph***	***Graphlet***	***Network Motif***	***Clique***	***Community (Module)***
Defining Characteristic	Generality: Any subset of nodes and edges from a graph. It is the foundational concept.	Topological Enumeration: A small, connected, and induced subgraph of a fixed size, used for systematic counting.	Statistical Significance: A subgraph that is significantly overrepresented in the network compared to a randomised null model.	Complete Connectivity: A subgraph where every node is directly connected to every other node (a complete graph).	Relative Density: A subgraph where nodes have a significantly higher density of connections internally than externally to the rest of the network.
Primary Analytical Goal	Isolation: To delimit a specific, known part of a network for focused study.	Structural Fingerprinting: To characterise and compare the local topology of nodes and networks by cataloguing all local connection patterns.	Identifying Functional Building Blocks: To discover recurring interaction patterns thought to be evolutionarily selected for specific biological functions.	Finding Core Functional Units: To identify maximally interconnected groups, often representing the stable core of protein complexes or a set of fully interdependent genes.	Revealing Modular Organisation: To partition the entire network into larger, functionally coherent modules that correspond to specific biological processes or pathways.

Typical Size	Arbitrary	Fixed & small (e.g., 2-5 nodes)	Small, commonly 3–5 nodes, occasionally larger.	Varies, but often small to medium (3-6 nodes) in biological contexts.	Varies widely, but generally larger than motifs and cliques.
Example in Biological Networks	A subnetwork representing the glycolysis pathway, manually extracted from a genome-scale metabolic network [107].	Using the distribution of 3, 4 and 5-node graphlets to classify the local structure of different PPI networks [108].	The feed-forward loop, an overrepresented motif in GRNs that can act as a signal filter. consisting of an input gene (X) that regulates an intermediate gene (Y), while both X and Y regulate a target gene (Z) [53].	A protein complex in a PPI network where every component physically interacts with every other component [109].	A module of co-expressed genes in a regulatory network that are all involved in the cellular response to stress [110].

References

1. Opsahl T, Agneessens F, Skvoretz J. Node centrality in weighted networks: Generalizing degree and shortest paths. Social networks. 2010;32: 245–251.

2. Kim H, Anderson R. Temporal node centrality in complex networks. Physical Review E. 2012;85: 026107.

3. Freeman LC. Centrality in social networks: Conceptual clarification. Social network: critical concepts in sociology Londres: Routledge. 2002;1: 238–263.

4. Iyer S, Killingback T, Sundaram B, Wang Z. Attack robustness and centrality of complex networks. PloS one. 2013;8: e59613.

5. Freeman LC. A set of measures of centrality based on betweenness. Sociometry. 1977; 35–41.

6. Jeong H, Mason SP, Barabási A-L, Oltvai ZN. Lethality and centrality in protein networks. Nature. 2001;411: 41–42.

7. Gallo G, Pallottino S. Shortest path methods: A unifying approach. Netflow at Pisa. 1986; 38–64.

8. Peyré G, Péchaud M, Keriven R, Cohen LD. Geodesic methods in computer vision and graphics. Foundations and Trends® in Computer Graphics and Vision. 2010;5: 197–397.

9. Bouttier J, Di Francesco P, Guitter E. Geodesic distance in planar graphs. Nuclear physics B. 2003;663: 535–567.

10. Hamed M, Spaniol C, Zapp A, Helms V. Integrative network-based approach identifies key genetic elements in breast invasive carcinoma. BMC genomics. 2015;16: 1–14.

11. Nazarieh M, Helms V. TopControl: A tool to prioritize candidate disease-associated genes based on topological network features. Scientific reports. 2019;9: 19472.

12. Nazarieh M, Wiese A, Will T, Hamed M, Helms V. Identification of key player genes in gene regulatory networks. BMC Systems Biology. 2016;10: 1–12.

13. Lavarenne J, Guyomarc'h S, Sallaud C, Gantet P, Lucas M. The spring of systems biology-driven breeding. Trends in plant science. 2018;23: 706–720.

14. Nacher JC, Akutsu T. Minimum dominating set-based methods for analyzing biological networks. Methods. 2016;102: 57–63.

15. Stadhouders R, Vidal E, Serra F, Di Stefano B, Le Dily F, Quilez J, et al. Transcription factors orchestrate dynamic interplay between genome topology and gene regulation during cell reprogramming. Nature genetics. 2018;50: 238–249.

16. Heinz S, Romanoski CE, Benner C, Glass CK. The selection and function of cell type-specific enhancers. Nature reviews Molecular cell biology. 2015;16: 144–154.

17. Boller S, Grosschedl R. The regulatory network of B-cell differentiation: a focused view of early B-cell factor 1 function. Immunological reviews. 2014;261: 102–115.

18. Schoenrock A, Burnside D, Moteshareie H, Pitre S, Hooshyar M, Green JR, et al. Evolution of protein-protein interaction networks in yeast. PLoS One. 2017;12: e0171920.

19. Lu X, Jain VV, Finn PW, Perkins DL. Hubs in biological interaction networks exhibit low changes in expression in experimental asthma. Molecular systems biology. 2007;3: 98.

20. Tummino PJ, Copeland RA. Residence time of receptor− ligand complexes and its effect on biological function. Biochemistry. 2008;47: 5481–5492.

21. Kusumi A, Tsunoyama TA, Suzuki KG, Fujiwara TK, Aladag A. Transient, nano-scale, liquid-like molecular assemblies coming of age. Current Opinion in Cell Biology. 2024;89: 102394.

22. Bagatell R, Whitesell L. Altered Hsp90 function in cancer: a unique therapeutic opportunity. Molecular cancer therapeutics. 2004;3: 1021–1030.

23. Wang W, Sreekumar PG, Valluripalli V, Shi P, Wang J, Lin Y-A, et al. Protein polymer nanoparticles engineered as chaperones protect against apoptosis in human retinal pigment epithelial cells. Journal of Controlled Release. 2014;191: 4–14.

24. Masquelier T, Thorpe SJ. Unsupervised learning of visual features through spike timing dependent plasticity. PLoS computational biology. 2007;3: e31.

25. Zeng G, Huang X, Jiang T, Yu S. Short-term synaptic plasticity expands the operational range of long-term synaptic changes in neural networks. Neural Networks. 2019;118: 140–147.

26. Kashyap G, Bapat D, Das D, Gowaikar R, Amritkar R, Rangarajan G, et al. Synapse loss and progress of Alzheimer's disease-A network model. Scientific Reports. 2019;9: 6555.

27. Holme P, Saramäki J. Temporal networks. Physics reports. 2012;519: 97–125.

28. Masuda N, Lambiotte R. A guide to temporal networks. World Scientific; 2016.

29. Carter SL, Brechbühler CM, Griffin M, Bond AT. Gene co-expression network topology provides a framework for molecular characterization of cellular state. Bioinformatics. 2004;20: 2242–2250.

30. Chaplin DD. Overview of the immune response. Journal of allergy and clinical immunology. 2010;125: S3–S23.

31. Kempe D, Kleinberg J, Kumar A. Connectivity and inference problems for temporal networks. 2000. pp. 504–513.

32. Zhang T, Gao Y, Qiu L, Chen L, Linghu Q, Pu S. Distributed time-respecting flow graph pattern matching on temporal graphs. World Wide Web. 2020;23: 609–630.

33. Van Der Wijst MG, de Vries DH, Brugge H, Westra H-J, Franke L. An integrative approach for building personalized gene regulatory networks for precision medicine. Genome medicine. 2018;10: 1–15.

34. Chan SS-K, Kyba M. What is a master regulator? Journal of stem cell research & therapy. 2013;3.

35. Itzkovitz S, Milo R, Kashtan N, Ziv G, Alon U. Subgraphs in random networks. Physical review E. 2003;68: 026127.

36. Ribeiro P, Paredes P, Silva ME, Aparicio D, Silva F. A survey on subgraph counting: concepts, algorithms, and applications to network motifs and graphlets. ACM Computing Surveys (CSUR). 2021;54: 1–36.

37. Bondy JA, Murty USR. Graph theory. Springer Publishing Company, Incorporated; 2008.

38. Estrada E, Rodriguez-Velazquez JA. Subgraph centrality in complex networks. Physical Review E—Statistical, Nonlinear, and Soft Matter Physics. 2005;71: 056103.

39. Estrada E, Rodríguez-Velázquez JA. Subgraph centrality and clustering in complex hyper-networks. Physica A: Statistical Mechanics and its Applications. 2006;364: 581–594.

40. Pržulj N, Corneil DG, Jurisica I. Modeling interactome: scale-free or geometric? Bioinformatics. 2004;20: 3508–3515.

41. Yaveroglu ON, Fitzhugh SM, Kurant M, Markopoulou A, Butts CT, Przulj N. ergm. graphlets: a package for ERG modeling based on graphlet statistics. arXiv preprint arXiv:14057348. 2014.

42. Milenković T, Pržulj N. Uncovering biological network function via graphlet degree signatures. Cancer informatics. 2008;6: CIN-S680.

43. Trpevski I, Dimitrova T, Boshkovski T, Stikov N, Kocarev L. Graphlet characteristics in directed networks. Scientific Reports. 2016;6: 37057.

44. Milo R, Itzkovitz S, Kashtan N, Levitt R, Shen-Orr S, Ayzenshtat I, et al. Superfamilies of evolved and designed networks. Science. 2004;303: 1538–1542.

45. Alon U. An introduction to systems biology: design principles of biological circuits. Chapman and Hall/CRC; 2019.

46. Alon U. Network motifs: theory and experimental approaches. Nature Reviews Genetics. 2007;8: 450–461.

47. Milo R, Shen-Orr S, Itzkovitz S, Kashtan N, Chklovskii D, Alon U. Network motifs: simple building blocks of complex networks. Science. 2002;298: 824–827.

48. Li Y, Lee KK, Walsh S, Smith C, Hadingham S, Sorefan K, et al. Establishing glucose- and ABA-regulated transcription networks in Arabidopsis by microarray analysis and promoter classification using a Relevance Vector Machine. Genome research. 2006;16: 414–427.

49. Lee TI, Rinaldi NJ, Robert F, Odom DT, Bar-Joseph Z, Gerber GK, et al. Transcriptional regulatory networks in Saccharomyces cerevisiae. science. 2002;298: 799–804.

50. Saddic LA, Huvermann B, Bezhani S, Su Y, Winter CM, Kwon CS, et al. The LEAFY target LMI1 is a meristem identity regulator and acts together with LEAFY to regulate expression of CAULIFLOWER. 2006.

51. Odom DT, Zizlsperger N, Gordon DB, Bell GW, Rinaldi NJ, Murray HL, et al. Control of pancreas and liver gene expression by HNF transcription factors. Science. 2004;303: 1378–1381.

52. Shen-Orr SS, Milo R, Mangan S, Alon U. Network motifs in the transcriptional regulation network of Escherichia coli. Nature genetics. 2002;31: 64–68.

53. Mangan S, Alon U. Structure and function of the feed-forward loop network motif. Proceedings of the National Academy of Sciences. 2003;100: 11980–11985.

54. McAdams HH, Shapiro L. Circuit simulation of genetic networks. Science. 1995;269: 650–656.

55. Ma'ayan A, Jenkins SL, Neves S, Hasseldine A, Grace E, Dubin-Thaler B, et al. Formation of regulatory patterns during signal propagation in a mammalian cellular network. Science. 2005;309: 1078–1083.

56. Apte AA, Cain JW, Bonchev DG, Fong SS. Cellular automata simulation of topological effects on the dynamics of feed-forward motifs. Journal of biological engineering. 2008;2: 1–12.

57. Kashtan N, Itzkovitz S, Milo R, Alon U. Topological generalizations of network motifs. Physical Review E—Statistical, Nonlinear, and Soft Matter Physics. 2004;70: 031909.

58. Piraveenan M, Wimalawarne K, Kasthurirathn D. Centrality and composition of four-node motifs in metabolic networks. Procedia Computer Science. 2013;18: 409–418.

59. Kepes F. Biological networks. World Scientific; 2007.

60. Yu S, Xu J, Zhang C, Xia F, Almakhadmeh Z, Tolba A. Motifs in big networks: Methods and applications. IEEE Access. 2019;7: 183322–183338.

61. Provan KG, Sebastian JG. Networks within networks: Service link overlap, organizational cliques, and network effectiveness. Academy of Management journal. 1998;41: 453–463.

62. Fadigas I de S, Pereira HB de B. A network approach based on cliques. Physica A: Statistical Mechanics and its Applications. 2013;392: 2576–2587.

63. Luce RD, Perry AD. A method of matrix analysis of group structure. Psychometrika. 1949;14: 95–116.

64. Baldwin NE, Chesler EJ, Kirov S, Langston MA, Snoddy JR, Williams RW, et al. Computational, integrative, and comparative methods for the elucidation of genetic coexpression networks. BioMed Research International. 2005;2005: 172–180.

65. Schmidt MC, Samatova NF, Thomas K, Park B-H. A scalable, parallel algorithm for maximal clique enumeration. Journal of parallel and distributed computing. 2009;69: 417–428.

66. Ouyang Q, Kaplan PD, Liu S, Libchaber A. DNA solution of the maximal clique problem. Science. 1997;278: 446–449.

67. Cazals F, Karande C. A note on the problem of reporting maximal cliques. Theoretical computer science. 2008;407: 564–568.

68. Cheng J, Ke Y, Fu AW-C, Yu JX, Zhu L. Finding maximal cliques in massive networks. ACM Transactions on Database Systems (TODS). 2011;36: 1–34.

69. Cheng J, Zhu L, Ke Y, Chu S. Fast algorithms for maximal clique enumeration with limited memory. 2012. pp. 1240–1248.

70. Abu-Khzam FN, Baldwin NE, Langston MA, Samatova NF. On the relative efficiency of maximal clique enumeration algorithms, with applications to high-throughput computational biology. 2005. pp. 1–10.

71. Wang J, Liu B, Li M, Pan Y. Identifying protein complexes from interaction networks based on clique percolation and distance restriction. BMC genomics. 2010;11: 1–14.

72. Albert R, Barabási A-L. Statistical mechanics of complex networks. Reviews of modern physics. 2002;74: 47.

73. Barrat A, Barthelemy M, Vespignani A. Dynamical processes on complex networks. Cambridge university press; 2008.

74. Bedru HD, Yu S, Xiao X, Zhang D, Wan L, Guo H, et al. Big networks: A survey. Computer Science Review. 2020;37: 100247.

75. Wasserman S, Faust K. Social network analysis: Methods and applications. 1994.

76. Intanagonwiwat C, Estrin D, Govindan R, Heidemann J. Impact of network density on data aggregation in wireless sensor networks. IEEE; 2002. pp. 457–458.

77. Bhattacharya S, Sinha S, Dey P, Saha A, Chowdhury C, Roy S. Online social-network sensing models. Computational Intelligence Applications for Text and Sentiment Data Analysis. Elsevier; 2023. pp. 113–140.

78. Jiang P, Singh M. SPICi: a fast clustering algorithm for large biological networks. Bioinformatics. 2010;26: 1105–1111.

79. Holman J. Dense graphlet statistics of protein interaction and random networks. Biocomputing 2009. World Scientific; 2009. pp. 178–189.

80. Golbeck J. Chapter 3 - Network Structure and Measures. In: Golbeck J, editor. Analyzing the Social Web. Boston: Morgan Kaufmann; 2013. pp. 25–44. doi:https://doi.org/10.1016/B978-0-12-405531-5.00003-1

81. Barabási A-L, Bonabeau E. Scale-free networks. Scientific american. 2003;288: 60–69.

82. Barabási A-L. Scale-free networks: a decade and beyond. science. 2009;325: 412–413.

83. Barabasi A-L, Oltvai ZN. Network biology: understanding the cell's functional organization. Nature reviews genetics. 2004;5: 101–113.

84. Adamic LA, Huberman BA. Power-law distribution of the world wide web. science. 2000;287: 2115–2115.

85. Zhao J, Xu K. Enhancing the robustness of scale-free networks. Journal of Physics A: Mathematical and Theoretical. 2009;42: 195003.

86. Cohen R, Erez K, Ben-Avraham D, Havlin S. Resilience of the internet to random breakdowns. Physical review letters. 2000;85: 4626.

87. Albert R, Jeong H, Barabási A-L. Error and attack tolerance of complex networks. nature. 2000;406: 378–382.

88. Xiao S, Xiao G, Cheng T, Ma S, Fu X, Soh H. Robustness of scale-free networks under rewiring operations. Europhysics Letters. 2010;89: 38002.

89. Cooper TF, Morby AP, Gunn A, Schneider D. Effect of random and hub gene disruptions on environmental and mutational robustness in Escherichia coli. Bmc Genomics. 2006;7: 1–11.

90. Li Y, Shang Y, Yang Y. Clustering coefficients of large networks. Information Sciences. 2017;382: 350–358.

91. Soffer SN, Vazquez A. Network clustering coefficient without degree-correlation biases. Physical Review E—Statistical, Nonlinear, and Soft Matter Physics. 2005;71: 057101.

92. Kong X, Shi Y, Yu S, Liu J, Xia F. Academic social networks: Modeling, analysis, mining and applications. Journal of Network and Computer Applications. 2019;132: 86–103.

93. Gilarranz LJ, Rayfield B, Liñán-Cembrano G, Bascompte J, Gonzalez A. Effects of network modularity on the spread of perturbation impact in experimental metapopulations. Science. 2017;357: 199–201.

94. Brandes U, Delling D, Gaertler M, Gorke R, Hoefer M, Nikoloski Z, et al. On modularity clustering. IEEE transactions on knowledge and data engineering. 2007;20: 172–188.

95. Malliaros FD, Vazirgiannis M. Clustering and community detection in directed networks: A survey. Physics reports. 2013;533: 95–142.

96. Newman ME, Girvan M. Finding and evaluating community structure in networks. Physical review E. 2004;69: 026113.

97. Ravasz E, Somera AL, Mongru DA, Oltvai ZN, Barabási A-L. Hierarchical organization of modularity in metabolic networks. science. 2002;297: 1551–1555.

98. Watts DJ, Strogatz SH. Collective dynamics of 'small-world'networks. nature. 1998;393: 440–442.

99. Lovejoy WS, Loch CH. Minimal and maximal characteristic path lengths in connected sociomatrices. Social Networks. 2003;25: 333–347.

100. Embar V, Handen A, Ganapathiraju MK. Is the average shortest path length of gene set a reflection of their biological relatedness? Journal of bioinformatics and computational biology. 2016;14: 1660002.

101. Doncheva NT, Kacprowski T, Albrecht M. Recent approaches to the prioritization of candidate disease genes. Wiley Interdisciplinary Reviews: Systems Biology and Medicine. 2012;4: 429–442.

102. Radivojac P, Peng K, Clark WT, Peters BJ, Mohan A, Boyle SM, et al. An integrated approach to inferring gene–disease associations in humans. Proteins: Structure, Function, and Bioinformatics. 2008;72: 1030–1037.

103. Zhang L, Li X, Tai J, Li W, Chen L. Predicting candidate genes based on combined network topological features: a case study in coronary artery disease. PloS one. 2012;7: e39542.

104. Köhler S, Bauer S, Horn D, Robinson PN. Walking the interactome for prioritization of candidate disease genes. The American Journal of Human Genetics. 2008;82: 949–958.

105. Masuda N, Sakaki M, Ezaki T, Watanabe T. Clustering coefficients for correlation networks. Frontiers in neuroinformatics. 2018;12: 7.

106. Rubinov M, Sporns O. Complex network measures of brain connectivity: uses and interpretations. Neuroimage. 2010;52: 1059–1069.

107. Faust K, Dupont P, Callut J, Van Helden J. Pathway discovery in metabolic networks by subgraph extraction. Bioinformatics. 2010;26: 1211–1218.

108. Pržulj N. Biological network comparison using graphlet degree distribution. Bioinformatics. 2007;23: e177–e183.

109. Bader GD, Hogue CW. An automated method for finding molecular complexes in large protein interaction networks. BMC bioinformatics. 2003;4: 2.

110. Dieckmann L, Cole S, Kumsta R. Stress genomics revisited: gene co-expression analysis identifies molecular signatures associated with childhood adversity. Translational psychiatry. 2020;10: 34.
